# Characterization of disease resistance genes in the *Brassica napus* pangenome reveals significant structural variation

**DOI:** 10.1111/pbi.13262

**Published:** 2019-10-10

**Authors:** Aria Dolatabadian, Philipp E. Bayer, Soodeh Tirnaz, Bhavna Hurgobin, David Edwards, Jacqueline Batley

**Affiliations:** ^1^ UWA School of Biological Sciences and the UWA Institute of Agriculture Faculty of Science The University of Western Australia Crawley WA Australia

**Keywords:** *Brassica napus*, pangenome, RGAugury, presence/absence variation, resistance gene

## Abstract

Methods based on single nucleotide polymorphism (SNP), copy number variation (CNV) and presence/absence variation (PAV) discovery provide a valuable resource to study gene structure and evolution. However, as a result of these structural variations, a single reference genome is unable to cover the entire gene content of a species. Therefore, pangenomics analysis is needed to ensure that the genomic diversity within a species is fully represented. *Brassica napus* is one of the most important oilseed crops in the world and exhibits variability in its resistance genes across different cultivars. Here, we characterized resistance gene distribution across 50 *B. napus* lines. We identified a total of 1749 resistance gene analogs (RGAs), of which 996 are core and 753 are variable, 368 of which are not present in the reference genome (cv. Darmor‐*bzh*). In addition, a total of 15 318 SNPs were predicted within 1030 of the RGAs. The results showed that core R‐genes harbour more SNPs than variable genes. More nucleotide binding site‐leucine‐rich repeat (NBS‐LRR) genes were located in clusters than as singletons, with variable genes more likely to be found in clusters. We identified 106 RGA candidates linked to blackleg resistance quantitative trait locus (QTL). This study provides a better understanding of resistance genes to target for genomics‐based improvement and improved disease resistance.

## Introduction

*Brassica napus* (canola/rapeseed/oilseed rape), belonging to the Brassicaceae family, is one of the three allotetraploid species in the triangle of U (UN, 1935) (AACC, *n* = 19). The species was formed ~7500 years ago through interspecific hybridization between the diploids *B. rapa* (Asian cabbage, turnip, AA genome) and *B. oleracea* (cabbage, cauliflower, Brussel sprouts, CC genome; Chalhoub *et al*., 2014). Canola is one of the most economically important oilseed crops in the world, grown mainly for its seeds, which yield between 35% and 45% edible oil.

With the advent of reference genome sequences, genomic approaches can be used to discover specific genes and subsequent association of candidate genes with heritable traits (Edwards *et al*., 2011; Qiu *et al*., 2013). However, a single reference genome cannot cover the entire gene content of a species due to structural variations, such as gene presence/absence variations (PAVs) or copy number variations (CNVs) (Gan *et al*., 2011; Golicz *et al*., 2016a; Hurgobin and Edwards, 2017). To address this issue, pangenomes have been constructed for a number of plant species, including maize, soya bean, rice, wheat and *Brassica* species (Golicz *et al*., 2016b; Hirsch *et al*., 2014; Hurgobin *et al*., 2018; Li *et al*., 2014; Lin *et al*., 2014; Montenegro *et al*., 2017; Yao *et al*., 2015).

The term ‘pangenome’ includes the complete and non‐redundant set of genes in the entire species; it is composed of core genes, which are present in all individuals, and variable genes, which are present only in some individuals (Golicz *et al*., 2016a; Hurgobin and Edwards, 2017). Variable genes can be split into two groups: CNVs, in which the number of copies of a gene differs between individuals, and PAVs, an extreme form of CNV in which a gene is present in some individuals but absent in others (Golicz *et al*., 2016a; Saxena *et al*., 2014). Golicz *et al*. (2016b) reported that in the *B. oleracea* pangenome, nearly 20% of genes are affected by presence/absence variation. The *Glycine soja* pangenome was analysed by Li *et al*. (2014) who identified 80% of the pangenome was present in all *G. soja* accessions, whereas the remainder was dispensable and displayed greater variation than the core genome. Therefore, the pangenome could serve as a valuable resource for scientists involved in crop genomics and breeding for understanding the diversity of genes and variations, for association with agronomic traits, including disease resistance, flowering time and yield.

To date, numerous resistance genes (R‐genes) have been discovered from many plant species. These plant resistance genes play specific roles in pathogen resistance. Resistance gene analogs (RGAs) can be grouped as either nucleotide binding site‐leucine‐rich repeats (NBS‐LRRs) or transmembrane leucine‐rich repeats (TM‐LRRs) (Sekhwal *et al*., 2015).

NBS‐LRR domain‐containing proteins are the largest family of R‐proteins (Dangl and Jones, 2001) and can be subdivided further into the TIR‐NBS‐LRR (TNL) and the non‐TIR‐NBS‐LRR (nTNL), which are distinguished by the presence of a Toll/Interleukin‐1 receptor (TIR) domain in the protein amino terminus (Shao *et al*., 2016; Zhou *et al*., 2004). Because most nTNL genes encode a coiled‐coil (CC) domain at the N terminus, the nTNL genes often are called CC‐NBS‐LRR (CNL) genes (Ameline‐Torregrosa *et al*., 2008; Shao *et al*., 2019). Likewise, TM‐LRRs can be subdivided into two classes: surface‐localized receptor‐like protein kinases (RLKs) and membrane‐associated receptor‐like proteins (RLPs) (Hammond‐Kosack and Jones, 1997). RLKs and RLPs are a large group of proteins that are necessary not only for regular plant development (Morris and Walker, 2003) but also for plant disease resistance (Kruijt *et al*., 2005). RLKs carry a cytoplasmic kinase domain, while RLPs carry a short cytoplasmic tail.

Sequencing of *Brassica* genomes has resulted in reference genome sequences for *B. napus* (Bayer *et al*., 2017; Chalhoub *et al*., 2014; Sun *et al*., 2018), *B. rapa* (Cai *et al*., 2017; Wang *et al*., 2011), *B. oleracea* (Liu *et al*., 2014; Parkin *et al*., 2014), *B. juncea* (Yang *et al*., 2016) and *B. nigra* (Yang *et al*., 2016), permitting a comprehensive study of R‐genes in these *Brassica* species. For example, Alamery *et al*. (2017) found 641, 443 and 249 NBS‐LRR genes in *B. napus*,* B. oleracea* and *B. rapa*, respectively, while Yu *et al*. (2014) identified 157, 206 and 167 NBS‐LRR genes in *B. oleracea*,* B. rapa* and *A. thaliana*, respectively, which may be due to using different approaches. Moreover, a total of 1989 RGA candidates were identified in the *B. oleracea* pangenome by Bayer *et al*. (2018).

The first objective of the current study was to identify RGAs in *B. napus* on a pangenome‐wide scale, to detect presence/absence variation. The second objective was to find morphotype‐specific RGAs and single nucleotide polymorphisms (SNPs) in the R‐genes to better understand the features of RGAs, such as numbers, distribution, variation, and physical locations in *B. napus*. Finally, the last objective was to find out whether the NBS genes differ among *B. napus* morphotypes. We also investigated whether genes in clusters are more likely to be lost or conserved. This work illustrates the value of pangenomes in disease resistance studies and identification of R‐genes. In addition, since only two R‐genes have been cloned in *B. napus* so far, this study may provide a platform to search for candidate *R*‐genes associated with disease resistance in this important crop.

## Results

### Genome‐wide analysis of RGAs

A total of 1749 RGAs were identified in the *B. napus* pangenome, comprising 503 NBS‐encoding genes and TX, 148 RLPs and 1098 RLKs. Of these 1749 genes, 996 (56.95%) were core (present in all lines) and 753 (43.05%) were variable. A total of 644 RGAs were on the A genome (493 core and 151 variable genes), 700 were on the C genome (484 core and 216 variable genes), 368 RGAs (all variable) were found in the pangenome additional contigs, and 37 RGAs were identified on the reference genome unplaced contigs (19 core and 18 variable; Table 1).

**Table 1:** The number of different RGA candidates and subfamilies found on the reference genomes, pangenome additional contigs and reference genome unplaced contigs

RGAs	Reference genome			Pangenome additional contigs	Reference genome unplaced contigs	Pangenome
	A genome	C genome	A and C			
CN	13 (4–9)*	5 (2–3)	18 (6–12)	10 (0–10)	1 (1–0)	29 (7–22)
CNL	3 (2–1)	10 (8–2)	13 (10–3)	17 (0–17)	0 (0–0)	30 (10–20)
NBS	10 (5–5)	20 (10–10)	30 (15–15)	43 (0–43)	2 (0–2)	75 (15–60)
NL	34 (19–15)	34 (20–14)	68 (39–29)	76 (0–76)	2 (2–0)	146 (41–105)
OTHER	7 (5–2)	12 (7–5)	19 (12–7)	6 (0–6)	2 (1–1)	27 (13–14)
RN	2 (2–0)	1 (0–1)	3 (2–1)	2 (0–2)	0 (0–0)	5 (2–3)
RNL	4 (3–1)	3 (0–3)	7 (3–4)	0 (0–0)	0 (0–0)	7 (3–4)
TN	8 (8–0)	7 (4–3)	15 (12–3)	12 (0–12)	0 (0–0)	27 (12–15)
TNL	12 (6–6)	18 (8–10)	30 (14–16)	13 (0–13)	0 (0–0)	43 (14–29)
TX	29 (12–17)	51 (25–26)	80 (37–43)	31 (0–31)	3 (0–3)	114 (37–77)
Total	122 (66–56)	161 (84–77)	283 (150–133)	210 (0–210)	10 (4–6)	503 (154–349)
RLP	37 (34–3)	39 (28–11)	76 (62–14)	70 (0–70)	2 (1–1)	148 (63–85)
RLK	485 (393–92)	500 (372–128)	985 (765–220)	88 (0–88)	25 (14–11)	1098 (779–319)
Total	522 (427–95)	539 (400–139)	1061 (827–234)	158 (0–158)	27 (15–12)	1246 (842–404)
Grand total	644 (493–151)	700 (484–216)	1344 (977–367)	368 (0–368)	37 (19–18)	1749 (996–753)

The numbers in parentheses represent the number of core and variable genes, respectively.

The numbers of different RGA candidates and subfamilies found on the reference genome, pangenome additional contigs and unplaced contigs are given in Table 1. In general, the NL and TX (TIR domain with unknown domain) subfamilies had the most members (146 and 114, respectively), while the RN and RNL subfamilies had the fewest members (Table 1). Of the 1344 RGAs identified on the reference genome, 283 were NBS‐encoding and TX genes (150 core and 133 variable genes), 76 were RLPs (62 core and 14 variable genes), and 985 were RLKs (765 core and 220 variable genes; Table 1).

A total of 368 R‐genes were not present in the reference genome assembly, including 210 NBS‐encoding and TX genes, 70 RLPs and 88 RLKs (Table 1). The majority were in the NL and NBS subfamilies (76 and 43, respectively), and the RN subfamily had the fewest members (2). No RNL genes were found in the pangenome additional contigs. There were also 25 RLKs, 2 RLPs and 10 NBS‐encoding and TX genes on the reference genome unplaced contigs (Table 1). In the pangenome, 73 270 RGAs were predicted across the 50 lines, 44 957 were found in the non‐synthetic, and 28,313 were found in the synthetic lines (Table 2).

**Table 2:** The total number of RGAs across the 50 lines on the reference genome, pangenome additional contigs and reference genome unplaced contigs

RGAs	Reference genomes			Pangenome additional contigs	Reference genome unplaced contigs	Pangenome
	A genome	C genome	A and C			
CN	602	241	843	156	50	1049
CNL	145	498	643	164	0	807
NBS	455	919	1374	635	94	2103
NL	1581	1611	3192	1147	100	4439
OTHER	332	563	895	19	99	1013
RN	100	49	149	31	0	180
RNL	199	142	341	0	0	341
TN	400	319	719	124	0	843
TNL	544	836	1380	129	0	1509
TX	1374	2332	3706	297	143	4146
Total	5732	7510	13 242	2702	486	16 430
RLP	1823	1928	3751	1380	98	5229
RLK	23 989	24 662	48 651	1748	1212	51 611
Total	25 812	26 590	52 402	3128	1310	56 840
Grand total	31 544	34 100	65 644	5830	1796	73 270
Non‐synthetics	19 727	21 256	40 983	2848	1126	44 957
Synthetics	11 817	12 844	24 661	2982	670	28 313

Within the reference genome, the 50 lines contained a total of 65 644 RGAs (40 983 in non‐synthetic lines with an average of 1322.03 RGAs per line and 24 661 in synthetic lines with an average of 1297.94 RGAs per line) with an average of 1312.88 RGAs per line, ranging from 1270 RGAs in H165 and R53 (both synthetic) to 1344 RGAs in Darmor (non‐synthetic; Table 2). The synthetic lines lost more RGAs (875 RGAs, with an average of 46.05 lost genes per line) than non‐synthetic lines (681 RGAs, with an average of 21.96 lost genes per line). The maximum (4.62%) and minimum (0.34%) gene losses were observed on chromosome A08 and chromosome C08, respectively (Table S1).

Within the reference genome unplaced contigs, there were 1796 RGAs, 1126 in non‐synthetic and 670 in synthetic lines (Table 2), ranging from 32 RGAs in G50 to 37 RGAs in S_39 (both synthetic lines). There were 54 RGAs (21 in non‐synthetic and 33 in synthetic lines) lost within the reference genome unplaced contigs. Similarly, the synthetic lines lost more RGAs than non‐synthetic lines (Table S1). The total numbers of different RGAs across the 50 lines on the chromosomes and reference genome unplaced contigs are presented in Table 2.

**Figure 1:**
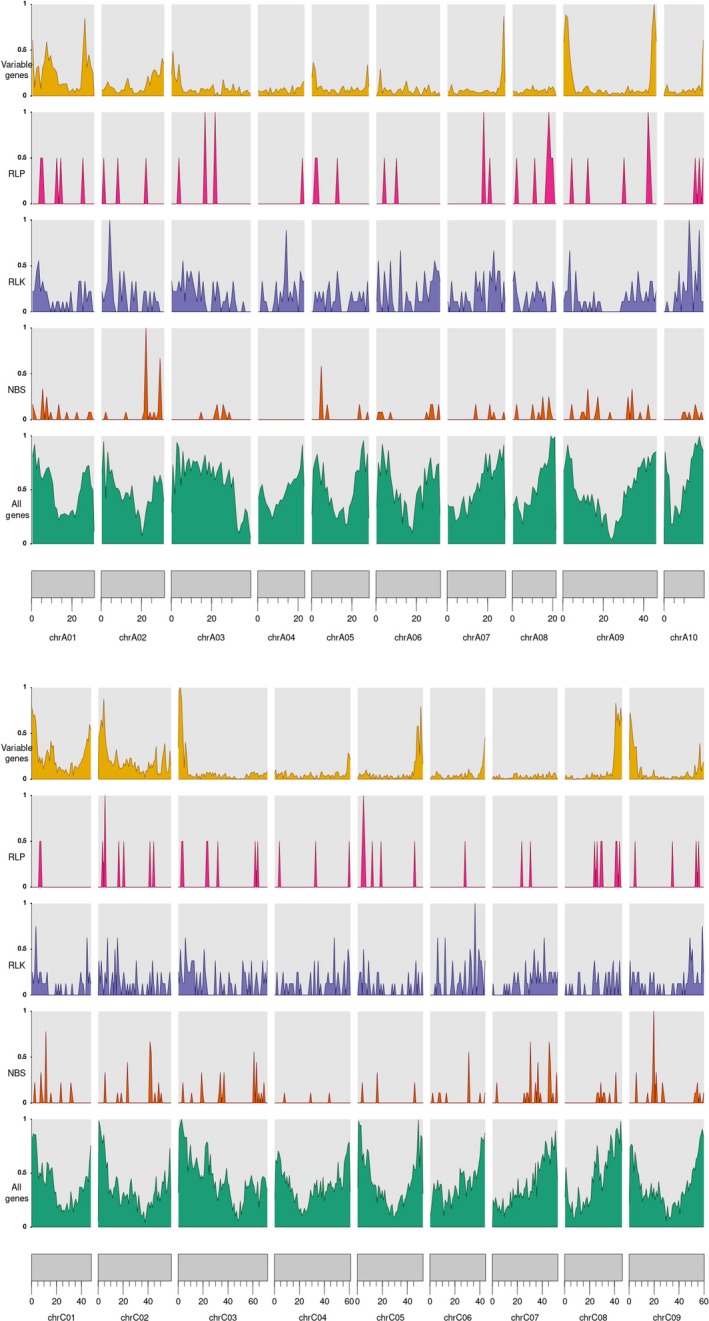
The distribution of variable genes and RLP, RLK and NBS domains across the reference genomes. These densities were normalized by the genome‐wide maximum of each measurement so that they peak at 1.

Within the pangenome additional contigs, the 50 lines contained a total of 5830 RGAs (2848 in non‐synthetic lines, with an average of 91.87 RGAs per line, and 2982 RGAs in synthetic lines, with an average of 156.94 RGAs per line), with an average of 116.6 RGAs per line, ranging from 24 RGAs in Darmor (non‐synthetic) to 209 RGAs in Cry_1 (synthetic) (Table 2 and Table S1). There were 21, 25 and 21 genes only identified in one, two and three lines, respectively. The total numbers of different RGAs across the 50 lines in the pangenome are given in Table 2. The distribution of different RGAs is given in Figure 1.

More TM‐LRR genes (78.94%) were predicted than NBS‐LRR and TX genes (21.06%). The numbers of different RGA candidates and subfamilies found on the reference genome are presented in Table 3. Out of the 1344 RGAs, 50 (3.72%) genes were typical or regular NBS‐LRR genes, with 30 TNLs (14 core and 16 variable), 13 CNLs (10 core and 3 variable) and 7 RNLs (3 core and 4 variable). The remaining 1294 RGAs (96.28%), known as non‐regular genes because of the lack of specific domains, were classified in nine groups of RLK (985), RN (3), RLP (76), TX (80), NL (68), NBS (30), CN (18), TN (15) and OTHER (19). RLKs and RLPs accounted for 73.29 and 5.65% of RGAs in the genome, respectively. The rest of the RGAs represented 17.85% (Tables 1 and 3).

**Table 3:** The number of different RGA candidates and subfamilies on the reference genome

Class	A01	A02	A03	A04	A05	A06	A07	A08	A09	A10	C01	C02	C03	C04	C05	C06	C07	C08	C09	**Total**
RLK	44 (23–21)*	50 (36–14)	65 (60–5)	41 (29–12)	35 (29–6)	64 (56–8)	54 (48–6)	32 (30–2)	55 (42–13)	45 (40–5)	43 (17–26)	60 (26–34)	87 (69–18)	55 (47–8)	35 (32–3)	60 (53–7)	60 (56–4)	35 (29–6)	65 (43–22)	985 (765–220)
RLP	5 (5–0)	3 (3–0)	5 (4–1)	1 (0–1)	3 (3–0)	2 (2–0)	3 (3–0)	7 (7–0)	5 (4–1)	3 (3–0)	2 (2–0)	7 (4–3)	7 (4–3)	3 (2–1)	6 (5–1)	1 (1–0)	2 (2–0)	7 (5–2)	4 (3–1)	76 (62–14)
RN	1 (1–0)	0 (0–0)	0 (0–0)	0 (0–0)	0 (0–0)	1 (1–0)	0 (0–0)	0 (0–0)	0 (0–0)	0 (0–0)	0 (0–0)	0 (0–0)	0 (0–0)	0 (0–0)	0 (0–0)	0 (0–0)	1 (0–1)	0 (0–0)	0 (0–0)	3 (2–1)
TNL	2 (0–2)	0 (0–0)	0 (0–0)	0 (0–0)	2 (0–2)	0 (0–0)	0 (0–0)	3 (1–2)	5 (5–0)	0 (0–0)	(1–0)	3 (1–2)	7 (2–5)	0 (0–0)	2 (2–0)	0 (0–0)	2 (1–1)	0 (0–0)	3 (1–2)	30 (14–16)
RNL	0 (0–0)	0 (0–0)	0 (0–0)	0 (0–0)	0 (0–0)	0 (0–0)	2 (2–0)	1 (1–0)	0 (0–0)	1 (0–1)	0 (0–0)	0 (0–0)	0 (0–0)	0 (0–0)	0 (0–0)	0 (0–0)	1 (0–1)	0 (0–0)	2 (0–2)	7 (3–4)
NL	3 (0–3)	12 (8–4)	4 (3–1)	0 (0–0)	4 (4–0)	1 (1–0)	2 (0–2)	3 (1–2)	5 (2–3)	0 (0–0)	5 (1–4)	2 (1–1)	6 (4–2)	0 (0–0)	1 (1–0)	1 (0–1)	8 (7–1)	4 (3–1)	7 (3–4)	68 (39–29)
NBS	2 (0–2)	2 (1–1)	1 (1–0)	0 (0–0)	1 (0–1)	1 (1–0)	0 (0–0)	1 (1–0)	1 (0–1)	1 (1–0)	2 (1–1)	5 (1–4)	4 (2–2)	0 (0–0)	0 (0–0)	1 (1–0)	2 (2–0)	2 (1–1)	4 (2–2)	30 (15–15)
CN	2 (0–2)	3 (2–1)	1 (0–1)	0 (0–0)	4 (1–3)	2 (0–2)	0 (0–0)	1 (1–0)	0 (0–0)	0 (0–0)	2 (0–2)	1 (1–0)	2 (1–1)	0 (0–0)	0 (0–0)	0 (0–0)	0 (0–0)	0 (0–0)	0 (0–0)	18 (6–12)
CNL	1 (0–1)	0 (0–0)	0 (0–0)	0 (0–0)	1 (1–0)	0 (0–0)	0 (0–0)	0 (0–0)	1 (1–0)	0 (0–0)	2 (1–1)	0 (0–0)	2 (2–0)	1 (1–0)	1 (1–0)	0 (0–0)	2 (2–0)	1 (1–0)	1 (0–1)	13 (10–3)
TN	0 (0–0)	1 (1–0)	1 (1–0)	0 (0–0)	0 (0–0)	1 (1–0)	0 (0–0)	0 (0–0)	3 (3–0)	2 (2–0)	0 (0–0)	0 (0–0)	0 (0–0)	1 (0–1)	0 (0–0)	2 (1–1)	2 (2–0)	1 (1–0)	1 (0–1)	15 (12–3)
OTHER	0 (0–0)	0 (0–0)	1 (0–1)	0 (0–0)	0 (0–0)	2 (1–1)	0 (0–0)	1 (1–0)	2 (2–0)	1 (1–0)	0 (0–0)	2 (1–1)	2 (0–2)	0 (0–0)	0 (0–0)	0 (0–0)	6 (4–2)	1 (1–0)	1 (1–0)	19 (12–7)
TX	6 (2–4)	8 (3–5)	0 (0–0)	0 (0–0)	0 (0–0)	3 (1–2)	2 (2–0)	2 (0–2)	6 (4–2)	2 (0–2)	7 (2–5)	11 (1–10)	5 (2–3)	1 (0–1)	3 (3–0)	7 (3–4)	8 (7–1)	1 (1–0)	8 (6–2)	80 (37–43)
Core	31	54	69	29	38	64	55	43	63	47	25	36	86	50	44	59	83	42	59	977
Variable	35	25	9	13	12	13	8	8	20	8	39	55	36	11	4	13	11	10	37	367
Core (%)	46.97	68.35	88.46	69.05	76.00	83.12	87.30	84.31	75.90	85.45	39.06	39.56	70.49	81.97	91.67	81.94	88.30	80.77	61.46	72.69
Variable (%)	53.03	31.65	11.54	30.95	24.00	16.88	12.70	15.69	24.10	14.55	60.94	60.44	29.51	18.03	8.33	18.06	11.70	19.23	38.54	27.31
Total	66	79	78	42	50	77	63	51	83	55	64	91	122	61	48	72	94	52	96	1344

The numbers in parentheses represent the number of core and variable genes, respectively.

The chromosomes C03 and A04 showed the maximum (122) and minimum (42) RGA numbers, respectively, whereas C07 contained the widest variety of RGA classes (11) and C04 showed the minimum (2) (Table 3). On average, there were 70.73 RGAs on each chromosome. No NBS‐encoding genes were identified on chromosome A04 in this study; there were only 41 RLKs and 1 RLP on chromosome A04. The absolute number of RGAs on chromosomes is illustrated in Figure S1.

The *B. napus* morphotype/lines that harbour at least one RGA of each class in the pangenome are presented in Table S2. TX, CNL and TN are not present in some non‐synthetic lines (Table S2). The class OTHER was only found in one non‐synthetic line (Nunsdale), showing that the class OTHER was more frequent in synthetic than non‐synthetic lines. The frequency of the RN across the synthetic and non‐synthetic lines was almost the same. There were no RNLs in the pangenome additional contigs. The non‐synthetic lines had less RGAs (2848 RGAs with an average of 91.87 RGAs per line) than synthetic lines (2982 RGAs with an average of 156.94 RGAs per line). A total of 12 570 RGAs were lost (8560 in non‐synthetics and 4010 in synthetic lines) across 50 lines within the pangenome additional contigs, with an average of 251.4 genes less per line, ranging from 159 in Cry_1 (synthetic) to 344 in Darmor (non‐synthetic) (Table S3). The non‐synthetic lines lost more RGAs (75.03%, average of 276.12 genes less per line) compared to the synthetics (57.35%, average of 211.05 genes less per line). There were 101 genes that were only found in synthetic lines and 3 genes that were only found in non‐synthetics. Only eight RGAs were present in all 50 lines (Table S1).

### SNP analysis

A total of 15 318 SNPs were identified within 1030 R‐genes: 10 584 SNPs within 731 core genes (70.97%) and 4734 SNPs within 299 variable genes (29.03%), with 719 R‐genes (265 core and 454 variable) containing no SNPs (Table S4). Out of the 1030 R‐genes carrying SNPs, 971 were on the reference genome (505 R‐genes on A genome, 396 core and 109 variable and 466 R‐genes on C genome, 324 core and 142 variable), 37 in the pangenome additional contigs (all variable) and 22 on reference genome unplaced contigs (11 core and 11 variable; Table S4). Out of the 15 318 SNPs, 14 467 (94.44%) were in the reference genome (7793 SNPs (50.87%) on the A genome and 6674 SNPs (43.56%) on the C genome), 594 (3.87%) were in the pangenome additional contigs, and 257 (1.67%) were on the reference genome unplaced contigs (Table S4). The maximum SNP number per gene was 131, with an average of 14.87 SNPs per gene carrying SNPs. The core R‐genes harboured more SNPs than variable genes. A total of 4943 SNPs were synonymous and 10 375 were non‐synonymous (Table 4). There were 1386 and 3557 synonymous SNPs and 3348 and 7027 non‐synonymous SNPs on variable and core R‐genes, respectively (Table S5).

**Table 4:** The number of SNPs and their effects in the reference genome, pangenome additional contigs and reference genome unplaced contigs

	Variants	Reference genome		A and C	Pangenome additional contigs	Reference genome unplaced contigs	Total number
		A genome	C genome				
Non‐synonymous	Variants_impact_HIGH	33	82	115	13	3	131
	Variants_impact_LOW	2963	2114	5077	174	80	5331
	Variants_impact_MODERATE	2015	2555	4570	245	98	4913
Sum		5011	4751	9762	432	181	10 375
Total non‐synonymous						10 375 (7027–3348) [Link]	
Synonymous	Variants_effect_synonymous_variant	2782	1923	4705	162	76	4943
Total synonymous						4943 (3557–1386)	
Total SNPs						15 318 (10 584–4734)	
Mis‐sense	Variants_effect_mis‐sense_variant	2015	2555	4570	245	98	4913
Total mis‐sense						4913 (3099–1814)	
Non‐sense	Variants_effect_stop_gained	18	46	64	5	1	70
	Variants_effect_stop_lost	4	5	9	3	1	13
	Variants_effect_stop_retained_variant	2	1	3	0	0	3
Sum				76	8	2	86
Total non‐sense						86 (48–38)	
Other effects	Variants_effect_5_prime_UTR_premature_start_codon_gain_variant	0	0	0	0	0	0
	Variants_effect_initiator_codon_variant	1	2	3	0	0	3
	Variants_effect_splice_acceptor_variant	1	18	19	2	1	22
	Variants_effect_splice_donor_variant	8	11	19	2	0	21
	Variants_effect_splice_region_variant	237	268	505	27	7	539
	Variants_effect_start_lost	2	2	4	1	0	5
Sum				550	32	8	590
Total						590 (422–168)	

The numbers in parentheses represent the number of SNPs on core and variable genes, respectively.

Of the non‐synonymous SNPs positioned within RGA candidates, 131 were predicted to be high impact (e.g. start or stop codon lost/gained), 5331 low impact (synonymous variant), and 4913 moderate impact (e.g. mis‐sense variant; Table 4). Of the high‐impact SNPs, 115 were in the reference genome (33 high impact on the A genome and 82 high impact on the C genome), 13 were in the pangenome additional contigs, and 3 were on the reference genome unplaced contigs. There were 5077 low‐impact SNPs in the reference genome (2963 low impact on the A genome and 2114 low impact on the C genome), 174 were in the pangenome additional contigs, and 80 were on the reference genome unplaced contigs (Table 4). Out of 4913 moderate‐impact SNPs, 4570 were in the reference genome (2015 moderate impact on the A genome and 2555 moderate impact on the C genome), 245 were in the pangenome additional contigs, and 98 were on the reference genome unplaced contigs (Table 4). Out of the 10 375 non‐synonymous SNPs, 5711 SNPs (55.04%) were transitions and 4,570 (44.04%) were transversions. There were 47 (0.45%) triallelic SNPs.

A total of 4913 variants were annotated as mis‐sense variants (3099 on core genes and 1814 on variable genes). In addition, 86 non‐sense variants (70 stop_gained, 13 stop_lost and 3 stop_retained_variant) were predicted on 73 genes (22 on A genome, 43 on C genome, 7 in the pangenome additional contigs and 1 on the reference genome unplaced contigs, within 40 core and 33 variable genes; Table 4).

A total of 3441 non‐sense variants (2825 stop_gained, 487 stop_lost and 129 stop_retained_variant) were detected across the 50 lines with an average of 68.82 non‐sense variants per line. The non‐synthetic lines contain 2233 non‐sense variants with an average of 72.03 per line, whereas synthetic lines contain 1208 with an average of 63.57 per line. The maximum (78) and minimum (49) non‐sense variant numbers were in Alaska (non‐synthetic) and MOY_4 (synthetic) lines, respectively. The distribution of non‐sense variants across the lines is illustrated in Figure S2.

**Figure 2:**
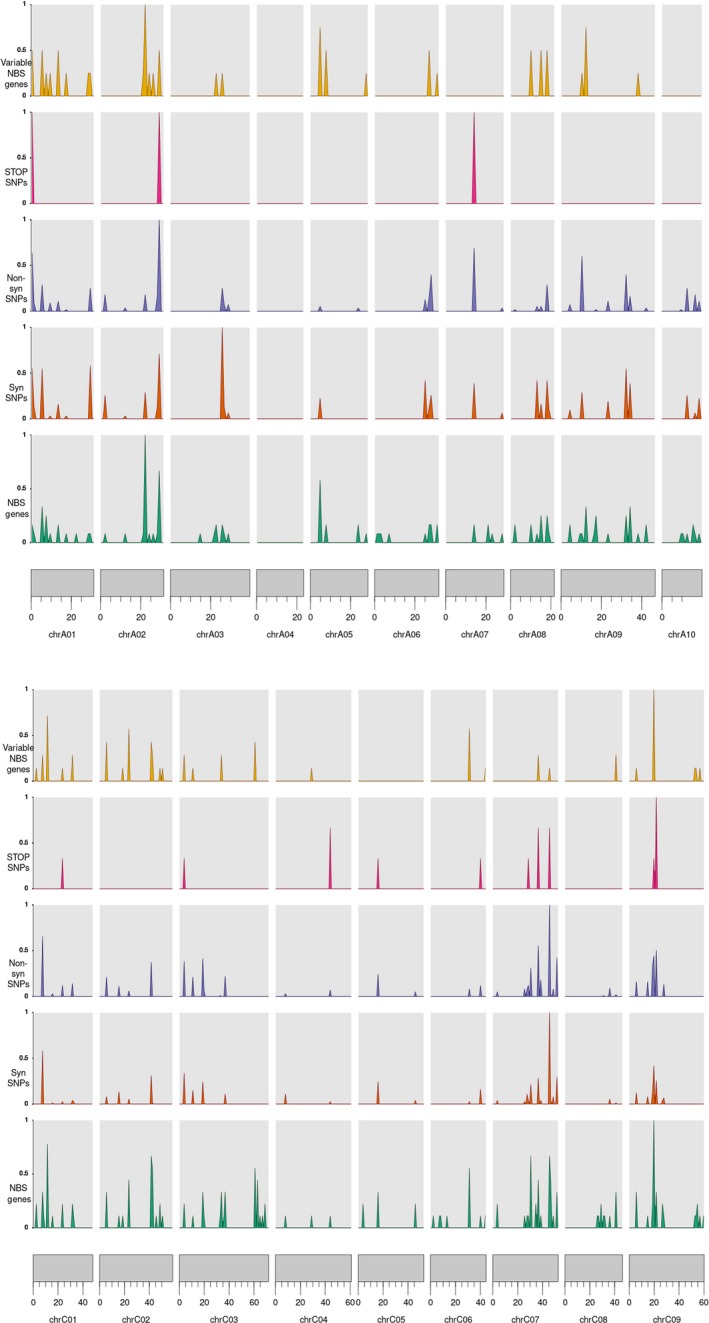
The density of NBS genes vs. variable NBS genes vs. STOP, synonymous and non‐synonymous SNPs across the reference genomes. These densities were normalized by the genome‐wide maximum of each measurement so that they peak at 1.

In summary, out of the 15 318 SNPs, 11 283 were in the TM‐LRR genes and 4035 were in the NBS‐LRR and TX genes (Table S6). Among the NBS‐LRR genes, NL and RN contained the maximum and minimum number of SNPs, respectively. RLKs harboured more SNPs than RLPs. All the RGA candidates were found to have more non‐synonymous SNPs than synonymous SNPs (Table S6). Also, variable R‐genes had a lower number of synonymous and non‐synonymous SNPs compared to core genes. However, the average SNP number per gene in variable genes was higher than core genes (15.83 vs. 14.47). The density of NBS genes vs. variable NBS genes vs. STOP, synonymous and non‐synonymous SNPs is illustrated in Figure 2.

### RGA clustering

#### NBS‐LRR clustering

NBS clustering analysis showed that 193 NBS‐encoding and TX genes (100 variable and 93 core genes) occurred within 62 clusters and 100 genes (39 variable and 61 core genes) were not clustered (Figures S3 and S4). Out of these 193 clustered NBS and TX genes, 39 genes harboured 622 SNPs (15.94 SNPs per gene; 6.44 SNPs per 1 kb). Also, out of 100 singletons, 66 genes carried 753 SNPs (11.40 SNPs per gene; 4.62 SNPs per 1 kb). Thus, genes in cluster showed more SNPs than singletons.

In the A genome, 77 genes were clustered in 24 clusters, and in the C genome, 112 genes were clustered in 36 clusters. Four unplaced genes were in 2 clusters. The average number of genes contained in a cluster in the genome was 3.1 genes, where the average in the A genome (3.2 genes) was found to be slightly larger than in the C genome (3.1 genes). The highest number of 7 clusters was found on chromosome C03, followed by 6 clusters on chromosomes C02, C07 and C09. The highest cluster number on the A genome was on chromosome A09 (5 clusters). No clusters were found on chromosome C04 (Figure S3). The highest gene number in a cluster was found on chromosome A02 with 12 genes, followed by chromosome C09 with 9 genes and chromosome A02 with 8 genes. There were more TNL clusters (6 clusters across the A genome and 12 clusters on the C genome) than CNL clusters (2 clusters on the A genome and 3 clusters on the C genome; Figures S3 and S4). There were no mixed clusters of TNLs and CNLs on the genome (Figures S3 and S4). A chi‐square test was performed to see whether ‘in cluster’ is dependent on ‘PAV’. With a *P*‐value of 0.04, the null hypothesis (saying two categories are independent) was rejected, so the two categories were found to be dependent; in other words, PAV and physical clustering of RGAs are linked. In general, variable genes were more likely to be found in clusters.

#### TM‐LRR clustering

When RLKs and RLPs were included, clustering analysis indicated that 640 genes (436 core genes and 204 variable genes) were clustered in 228 clusters, with an average of 2.80 genes per cluster (Figures S5 and S6). Out of these 640 clustered genes, 442 genes harboured 4873 SNPs (11.02 SNPs per gene; 4.38 SNPs per 1 kb). In the A genome, 291 genes were clustered in 103 clusters, and in the C genome, 341 genes were clustered in 122 clusters. The average number of genes in a cluster in the C genome (2.79 genes) was found to be smaller than in the A genome (average 2.82 genes). Out of 228 clusters, the highest number was 28 clusters found on C03 followed by 16 clusters on C02 and C09 (Figures S5 and S6). The highest gene number in a cluster was found on A02 with 13 genes followed by C02 with 9 genes and C09 with 9 genes (Figures S5 and S6).

There were 90 and 13 clusters on the A genome containing RLK and RLP genes, respectively. Furthermore, there were 12, 5, 96 and 15 clusters on the C genome carrying TNL, CNL, RLK and RLP genes, respectively (Figures S5 and S6). Overall, there were more RLK clusters (186) than RLP (28), TNL (18) and CNL (7) clusters in the genome.

#### Linking known QTL and R‐genes

**Figure 3:**
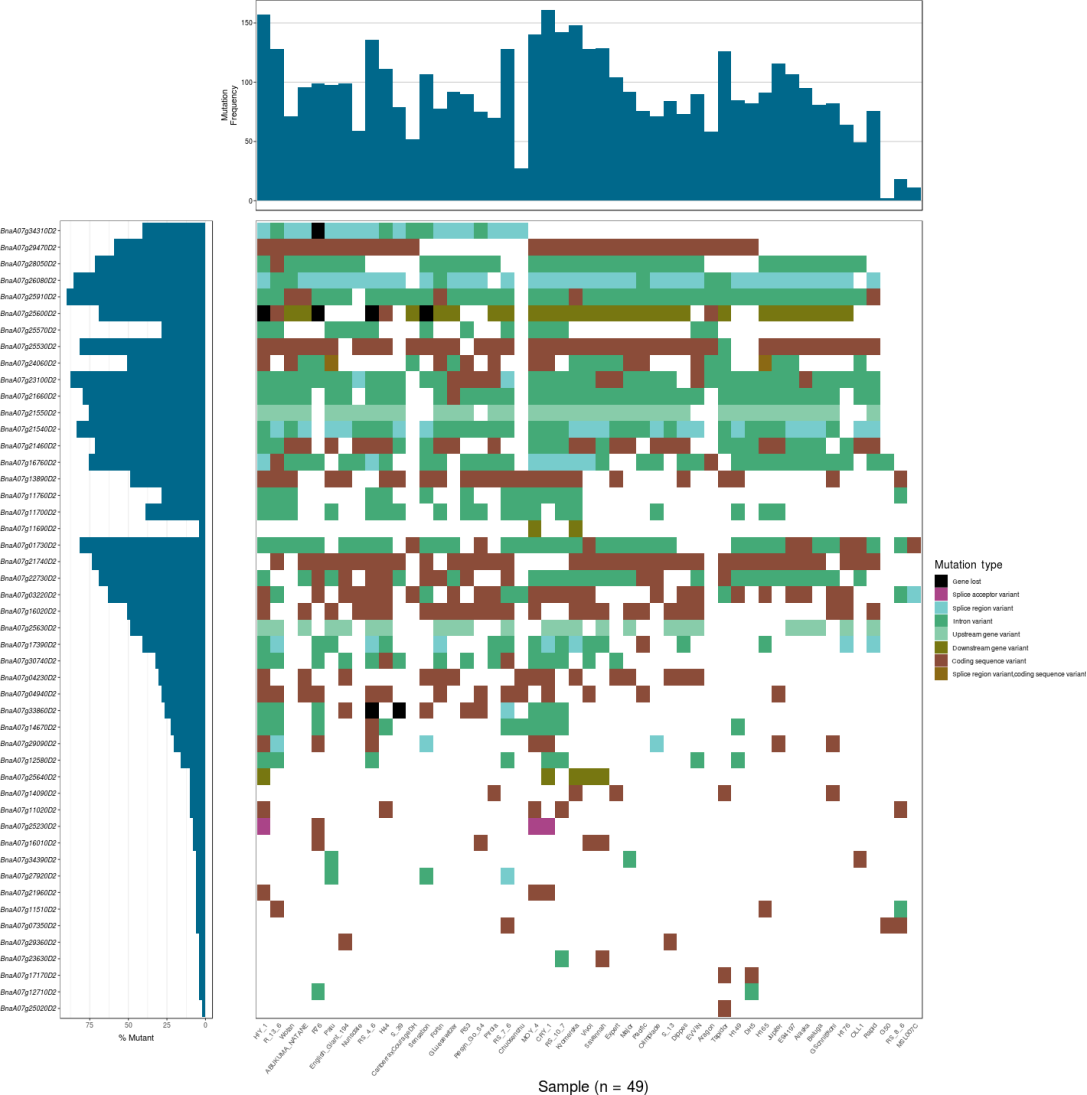
Waterfall plot of the blackleg‐linked QTL (*Rlm4* locus). Gene order is determined by the position in the reference assembly.

The RGA candidate positions were compared with known quantitative trait loci (QTL) for blackleg resistance to identify possible candidate genes. Positions were predicted for 32 QTL markers (Table S7) from genetic mapping of seven loci; *LepR1* (A02), *LepR2* (A10), *Rlm1*,* Rlm3*,* Rlm4*,* Rlm7* and *Rlm9* (A07) in the Darmor‐*bzh* v 8.1 assembly. For each locus, if there was more than one known QTL, the QTL were combined to create a single region, the QTL name and references are shown in Table S7. *Rlm1* was localized within an interval of approximately 4.94 Mbp containing 18 RGAs. The mapping of *Rlm3* and *Rlm4* placed these R loci within intervals of 16.79 Mb (46 genes) and 26.69 Mbp (60 genes), respectively. *Rlm7* and *Rlm9* loci were localized within 16.02 (46 genes) and 5.35 Mbp (17 genes), respectively. The A02 (*LepR1*) and A10 (*LepR2*) R‐genes were localized to regions 10.41 (14 genes) and 13.95 Mbp (32 genes). *Rlm1* and *Rlm9* were in the narrowest QTL, which covered 18 (16 core and 2 variable) and 17 (all core) genes, respectively (Table S7). Overlapping *Rlm1*,* Rlm3*,* Rlm4*,* Rlm7* and *Rlm9* were combined into single, contiguous non‐redundant regions. Figure S7 provides an illustration of how these QTL were combined. There were 60 (53 core and 7 variable) RGAs within *Rlm1*,* Rlm3*,* Rlm4*,* Rlm7* and *Rlm9* on chromosome A07, 14 (12 core and 2 variable) RGAs within *LepR1* on chromosome A02 and 32 (28 core and 4 variable) RGAs within *LepR2* on chromosome A10 (Table S7). We identified 688 SNPs in these 106 RGAs (70 within *LepR1*, 180 within *LepR2* and 438 within *Rlm1*,* Rlm3*,* Rlm4*,* Rlm7* and *Rlm9)*. The RGA classes showed different levels of variability; for example, on chromosome A07, out of 60 RGAs within the QTL, only 5 RLKs and 2 NL were variable. Also, on chromosome A02, out of 14 RGAs, only 2 RLKs were variable, while on chromosome A10, 4 variable RLKs were found out of 29 RLKs (Table S7). A waterfall plot of the blackleg resistance‐linked QTL (*Rlm4* locus) was produced to show the mutational load of RGA candidates located within the QTL candidate region in all the individuals (Figure 3). CRY_1 showed the highest mutational load followed by HIY_1. Three genes (*BnaA07g34310D2*,* BnaA07g25600D2* and *BnaA07g33860D2*) were lost in five individuals (HIY_1, R76, RS_4_6, S_39 and sensation). Also, the maximum and minimum mutation percentages were related to *BnaA07g25910D2* and *BnaA07g25020D2* genes, respectively. Only *BnaA07g24060D2* showed a splice region variant and coding sequence variant in two individuals (Palu and H165). A splice acceptor variant was only observed in the individuals HIY_1, CRY_1 and MOY_4 in the gene *BnaA07g25230D2*. The coding sequence variant was the only mutation that was detected in all individuals.

## Discussion

The sequencing and assembly of *Brassica* genomes have allowed tremendous progress in genotyping and gene identification; however, to date, only two R‐genes have been cloned in *B. napus* despite extensive work. Therefore, there is a need to perform analyses to overcome this limitation. As a sequenced reference cultivar does not contain all *B. napus* genes due to gene presence/absence or copy number variation between individuals, we used the *B. napus* pangenome for the identification and characterization of RGA candidates (NBS‐LRR and TM‐LRR), which are not present in the single reference assembly, to examine their distribution, domain structure, clustering, presence/absence and SNPs. Our results show that a pangenome is essential to identify candidate genes for breeding of improved cultivars. The findings can be exploited to further characterize the relationships between candidate R‐genes and resistance/susceptibility among cultivars. In general, the results demonstrated that the RGAs were unevenly distributed across the genomes. This observation has also been noted in other species, where an uneven distribution of RGAs on chromosomes appears to be common in plants (Kohler *et al*., 2008; Meyers *et al*., 2003; Porter *et al*., 2009; Yang *et al*., 2008; Zhou *et al*., 2004). This uneven distribution might be due to recent tandem gene amplifications, segmental duplications (Rice Chromosomes 11 and 12 Sequencing Consortia, 2005) and dosage compensation (Zhu *et al*., 2018). Gene dosage balance is critical for development and phenotypic characteristics, especially in synthetic *B. napus* individuals at initial generations (Xiong *et al*., 2011). The results suggest that gene order and proximity are important for the functional nature of these genes (Singh *et al*., 2015).

TNLs were found to be more abundant than CNLs. The greater number of TNLs compared with CNLs has been previously reported in *B. napus* (Alamery, 2015; Alamery *et al*., 2017), *B. rapa*,* Arabidopsis* (Meyers *et al*., 2003; Mun *et al*., 2009; Yu *et al*., 2014) and *Linum usitatissimum* (linseed) (Kale *et al*., 2013). It has been reported that the TIR domain is an important component of innate immunity across species through self‐association and ligand‐specific protein–protein interactions (Ve *et al*., 2015). Leister (2004) stated that the over‐representation of one of these families could reflect the adaptation of the R‐genes to the predominant pathogens. The variation in NBS‐LRR gene content between individuals has been assumed to play an important role in the resistance or susceptibility of crops to disease (Tollenaere *et al*., 2012; Wu *et al*., 2014). Furthermore, we found that the largest class of RGA candidates were RLKs, which has been previously reported in other plants, such as wild strawberry and cotton (Chen *et al*., 2015; Li *et al*., 2017). This frequency might be due to a greater diversity of roles of RLK than RLP and NBS‐LRR genes.

The core R‐genes not only had more total SNPs than variable R‐genes, but also had a higher number of synonymous and non‐synonymous SNPs. In a previous study, Bayer *et al*. (2018) reported that in *B. oleracea,* core genes had more low‐ and moderate‐impact SNPs than variable genes, while core genes and variable genes showed almost identical numbers of high‐impact SNPs. Our result is different from that previously reported in soya bean, where the variable genes in the soya bean pangenome have a higher proportion of SNPs (Li *et al*., 2014). It has been reported that a higher proportion of non‐synonymous SNPs in the variable genes suggest a higher evolutionary rate of variable genes (Li *et al*., 2014).

In the present study, out of 9762 non‐synonymous SNPs detected in RGAs on the reference genomes, 5,011 were on the A genome and 4,751 were on the C genome. Similar results were found by Huang *et al*. (2013) who identified 55% of SNPs on the A genome and 45% of SNPs on the C genome. Similarly, Bancroft *et al*. (2011) identified 15 559 SNPs on the A genome and 5675 SNPs on the C genome. Uneven distribution of SNPs throughout the Brassica species genome is a common phenomenon and has also been reported in *A. thaliana* (Feltus *et al*., 2004). For instance, in *Arabidopsis*, R‐genes are known to accumulate large numbers of non‐synonymous SNPs, producing new allelic variants of R‐genes (Bakker *et al*., 2006). A higher proportion of non‐synonymous SNPs have been reported in other plants, for instance 56% non‐synonymous SNPs in oil palm (Pootakham *et al*., 2015), 57% in sorghum (Zheng *et al*., 2011) and 54%–57% in rice (Jeong *et al*., 2013; Subbaiyan *et al*., 2012).

Among the R‐genes, transition substitutions were more predominant than transversions. The increased frequency of transition substitutions in coding regions is likely due to the structure of the genetic code and selective constraints (Wondji *et al*., 2007). In addition, the higher frequency of transition SNPs may be partly related to 5‐methylcytosine deamination reactions that frequently occur, particularly at CpG dinucleotides (Holliday and Grigg, 1993).

Among the 12 RGA subfamilies, all had SNPs, suggesting that SNPs in the RGAs may alter protein interactions. It should be noted that SNPs located in different domains could be responsible for the differences in blackleg resistance. For example, SNPs might affect nucleotide binding in the NBS‐LRR domains and thus gene regulation and R‐protein function. Therefore, these RGAs can be selected as candidate genes for further characterization of RGA functional involvement in resistance to diseases and the development of molecular markers.

Many R‐genes often cluster in the genome (Hulbert *et al*., 2001). Previous studies have revealed that the majority of NSB‐LRR genes are present in gene clusters in plant genomes (Hulbert *et al*., 2001) conferring different resistance specificities (Leister, 2004). For example, in rice and *Arabidopsis*, 71% and 76% of NBS‐LRR genes were located within RGA gene‐rich clusters, respectively (Guo *et al*., 2011; Zhou *et al*., 2004). In addition, *Yr* genes responsible for resistance against wheat yellow rust were found to be clustered (Marchal *et al*., 2018). NBS‐LRR‐encoding genes are frequently clustered in the genome, as a result of both segmental and tandem duplications (Leister, 2004). It has been reported that R‐genes within a cluster can have different rates and patterns of variation, leading to the discrimination of two types of R‐genes based on their modes of evolution (Kuang *et al*., 2004). The maize *Rp1* cluster (∼1–52 homologs per haplotype (Smith *et al*., 2004)) and the lettuce *Dm3* (aka *RGC2*) cluster (∼12–32 homologs per haplotype (Kuang *et al*., 2004)) are among the largest R‐gene clusters. The role of R‐gene clusters in R‐gene evolution is often conceptualized in terms of a gene‐for‐gene model (Friedman and Baker, 2007). Individual clusters may also confer specific resistance to a wide range of different pathogens (van der Vossen *et al*., 2000). No mixed clusters of TNLs and CNLs were found in the *B. napus* pangenome. The separate clustering of TIR and non‐TIR‐NBS‐LRR sequences may be contributed to the ancient divergence of these two subfamilies, for example, by restricting locally acting mechanisms for sequence homogenization such as unequal crossing over (Zhu *et al*., 2002). The current results revealed that the NBS‐LRR and TM‐LRR classes are abundant and widely distributed throughout the genome and NBS‐LRR variable genes were more likely to be found in clusters. Thus, gene clustering may be a crucial attribute of the generation of novel resistance specificities through gene duplication or recombination (Meyers *et al*., 2003).

*Brassica napus* synthetic lines can be produced through crossing between parental species (*B. rapa* and *B. oleracea*) followed by embryo rescue and chromosome doubling. The performance and disease resistance of synthesized lines can be compared with non‐synthetic or parental lines. It has been shown that synthesized lines, especially early generations, are specifically prone to homoeologous rearrangements, including deletions, duplications and translocations (Gaeta *et al*., 2007; Hurgobin *et al*., 2018; Szadkowski *et al*., 2010; Xiong *et al*., 2011), also aneuploidy, gross chromosomal rearrangements and dosage balance mechanisms that enforce chromosome number stability (Xiong *et al*., 2011). Several studies have indicated that genetic changes caused by homoeologous chromosome rearrangement are common in newly resynthesized *B. napus* allotetraploids (Gaeta *et al*., 2007; Song *et al*., 1995). In this study, the synthetic lines were shown to exhibit more RGAs than non‐synthetic lines, whereas non‐synthetics lines have lost more RGAs, making them an interesting model to study the impact of polyploidization on genome structure, disease resistance genes and their potential associated with agronomic traits. Greater genetic diversity in synthetic *B. napus* lines compared with non‐synthetic lines has previously been reported (Golicz *et al*., 2016b; Hurgobin *et al*., 2018; Li *et al*., 2014). This difference has been attributed to the incorporation of novel alleles from diverse progenitor genomes and highlights the potential of using synthetic *B. napus* accessions as a source of novel genetic structural variation for breeding improved varieties (Hurgobin *et al*., 2018).

Several QTL responsible for quantitative resistance have been identified in *B. napus* cultivars (Huang *et al*., 2016; Jestin *et al*., 2011; Larkan *et al*., 2016; Raman *et al*., 2012b). We identified 106 RGA candidates and 688 SNPs within QTL regions associated with blackleg resistance. However, these were the larger QTL regions that could be narrowed in future analysis. Identification of RGA candidates within QTL may inform future breeding efforts in *B. napus* through providing a basis for mapping candidate genes, as markers linked to resistance are useful for understanding mechanisms of resistance and immediate breeding applications. The finding of both core and variable genes within these regions highlights the requirement of pangenomics in these efforts.

## Conclusion

In this study, we analysed RGAs in a *B. napus* pangenome using a single reference and whole‐genome sequencing data from 50 lines. We found that the presence of RGA candidates varies between lines and suggest that in *B. napus*, SNPs and presence/absence variation drive RGA diversity. Also, the results demonstrated chromosome imbalance in terms of PAV and SNPs. A genome contained less RGAs but more SNPs compared with C genome. This study emphasizes the value of analysing the pangenome in finding novel RGAs not contained within a single reference. Our results also highlight the potential of variable genes and synthetic lines to be used in genetic structural variation studies for future breeding programmes. Overall, the findings can be exploited to further characterize the relationships between RGAs and resistance/susceptibility among *B. napus* lines.

## Experimental procedures

### Pangenome

The *B. napus* pangenome, consisting of 31 non‐synthetic (3 fodders, 2 swedes, 2 vegetables and 24 oilseeds) and 19 synthetic accessions, was described in Hurgobin *et al*. (2018). The pangenome size was 1044 Mbp and contained 1749 predicted R‐genes. Gene PAV discovery was performed using the SGSGeneLoss package (Golicz *et al*., 2015) as described in Hurgobin *et al*. (2018) and Bayer *et al*. (2018).

### Identification of candidate *R*‐genes

The RGAugury pipeline (v 2017‐10‐21; Li *et al*., 2016) was used to automate RGA (NBS, RLK and RLP candidate genes) prediction in the *B. napus* Darmor‐*bzh* v8.1 annotation, downloaded from http://brassicagenome.net. The RGA candidates were classified into 12 subfamilies. The TM‐LRR was divided into RLP and RLK, and the NBS‐LRR candidates were divided based on the presence or absence of specific domains: Proteins carrying only an NB‐ARC domain were classified as NBS, proteins carrying TIR, NB‐ARC and leucine‐rich repeat domains were classified as TNLs, or TN if the leucine‐rich repeat domain was missing. Proteins carrying coils, NB‐ARC and leucine‐rich repeat domains were classified as CNLs, or CN if the leucine‐rich repeat domain was missing, or NL if the coils domain was missing. Proteins carrying a TIR domain with additionally unknown domains were classified as TX (The TX genes do no encode NBS domain and not all TX genes are derived from TNL genes). Proteins with NL genes with an RPW8 domain were classified as RNL, or RN if the leucine‐rich repeat domain was missing, while all other combinations (e.g. CNL + RPW8) were classified as OTHER.

### SNP discovery

Single nucleotide polymorphisms were previously predicted by Hurgobin *et al*. (2018). SNPEff v4.3T (Cingolani *et al*., 2012) was used for the variant effect prediction. The SNP impacts were predicted as high (to have a high, disruptive impact in the protein, probably causing protein truncation, loss of function or triggering non‐sense‐mediated decay), moderate (a non‐disruptive variant that might change protein effectiveness) and low to be mostly harmless or unlikely to change protein behaviour. Since there were more core than variable genes and core genes were longer than variable genes (Hurgobin *et al*., 2018), the counts of low‐, moderate‐, and high‐impact SNPs were normalized by dividing by the total length of all exons per gene in order to account for very long and very short genes. Two‐way ANOVA as implemented in R v3.4.2 (R Core Team, 2016) was used to check whether the variation in low, moderate, high, and upstream and downstream variants could be explained by the presence/absence status or by the RGA class.

### Physical clustering

Resistance gene analog clusters were determined by gene order on each chromosome (Holub, 2001; Meyers *et al*., 2003; Richly *et al*., 2002). RGA candidates were continuously merged into clusters if they were within 10 RGA or non‐RGA genes (makeRGeneClusterAnalysis.py). Physical clusters and presence/absence status were compared using Pearson's chi‐square test with Yates’ continuity correction as implemented in R v3.4.2 (R Core Team, 2016).

### Linking known QTL and R‐genes

Known blackleg resistance‐linked QTL were collected from the literature (Delourme *et al*., 2004; Larkan *et al*., 2016; Leflon *et al*., 2007; Raman *et al*., 2012a,b; Tang and Zhao, 2015). The sequences of the markers, genes and primer pairs were downloaded from the collected literature. BLAST was used to assign positions for the forward and reverse primer sequences in the v8.1 *B. napus* Darmor‐*bzh* assembly.

### Plots and graphs

The SNP and RGA density/distribution plots were generated using karyoploteR v1.4.2 (Gel and Serra, 2017). Waterfall plots were drawn using Variant Effect Predictor v88.13 (McLaren *et al*., 2016), GenVisR v1.11.3 (Skidmore *et al*., 2016), vcftools v0.1.15 (Danecek *et al*., 2011) and R 3.4.4 (R Core Team, 2016).

## Conflict of interest

All authors declare that they have no conflict of interest in relation to this publication.

## Author contributions

AD, PB, DE and JB conceived and designed the experiments. AD, PB, ST and BH performed the experiments and analysed the data. AD, PB, DE and JB wrote the paper.

## Supporting information


**Figure S1** The absolute number of RGAs on the reference genomes.
**Figure S2** The distribution of stop codons (stop_gained, stop_lost and stop_retained_variant) across the 50 non‐synthetic (fodder (blue), swede (red), vegetable (green) and oilseed (grey)) and synthetic (black) lines on the reference genome, pangenome additional contigs and reference genome unplaced contigs.
**Figure S3** Physical clustering of NBS‐LRR genes on the chromosome of the A genome of *B. napus*. The colourful circles above (variable) and below (core) each chromosome (grey bars) are designated for NBS classes. Chromosome lengths are shown in megabase pairs on the scale at the top.
**Figure S4** Physical clustering of NBS‐LRR genes on the chromosome of the C genome of *B. napus*. The colourful circles above (variable) and below (core) each chromosome (grey bars) are designated for NBS classes. Chromosome lengths are shown in megabase pairs on the scale at the top.
**Figure S5** Physical clustering of TM‐LRR genes on the chromosome of the A genome of *B. napus*. The colourful circles above (variable) and below (core) each chromosome (grey bars) are designated for NBS classes. Chromosome lengths are shown in megabase pairs on the scale at the top.
**Figure S6** Physical clustering of TM‐LRR genes on the chromosome of the C genome of *B. napus*. The colourful circles above (variable) and below (core) each chromosome (grey bars) are designated for NBS classes. Chromosome lengths are shown in megabase pairs on the scale at the top.
**Figure S7** *Rlm1*,* Rlm3*,* Rlm4*,* Rlm7* and *Rlm9* QTL were combined into non‐redundant QTL regions by combining QTL length overlaps into single contiguous regions. Non‐QTL regions contain no QTL whatsoever.

**Table S1** RGAs across the 50 lines on the A genome, C genome, pangenome additional contigs and reference genome unplaced contigs.

**Table S2** Morphotype/lines that harbour at least one RGA of each class in the pangenome.
**Table S3** The number of lost genes in the pangenome additional contigs, reference genome and reference genome unplaced contigs.
**Table S4** The number of SNPs in core and variable genes in the reference genome, pangenome additional contigs and reference genome unplaced contigs.
**Table S5** The numbers of non‐synonymous, synonymous and total SNPs on core and variable R‐genes.
**Table S6** The numbers of non‐synonymous and synonymous SNPs, mis‐sense and non‐sense variants and other effects in different RGAs.
**Table S7** RGA candidates underlying reported QTL for blackleg in the Darmor v 8.1 assembly.

## References

[pbi13262-bib-0001] Alamery, S.F. (2015). Genome‐wide identification of NBS‐LRR genes in Brassica and their association with disease resistance in Brassica napus. PhD Thesis. The University of Queensland. School of Agriculture and Food Sciences.

[pbi13262-bib-0002] Alamery, S. , Tirnaz, S. , Bayer, P. , Tollenaere, R. , Chaloub, B. , Edwards, D. and Batley, J. (2017) Genome‐wide identification and comparative analysis of NBS‐LRR resistance genes in *Brassica napus* . Crop Pasture Sci. 69, 79–93.

[pbi13262-bib-0003] Ameline‐Torregrosa, C. , Wang, B.B. , O'Bleness, M.S. , Deshpande, S. , Zhu, H. , Roe, B. , Young, N.D. *et al* (2008) Identification and characterization of nucleotide‐binding site‐leucine‐rich repeat genes in the model plant *Medicago truncatula* . Plant Physiol. 146, 5–21. 17981990 10.1104/pp.107.104588 PMC2230567

[pbi13262-bib-0004] Bakker, E.G. , Toomajian, C. , Kreitman, M. and Bergelson, J. (2006) A genome‐wide survey of R gene polymorphisms in *Arabidopsis* . Plant Cell, 18, 1803–1818. 16798885 10.1105/tpc.106.042614 PMC1533970

[pbi13262-bib-0005] Bancroft, I. , Morgan, C. , Fraser, F. , Higgins, J. , Wells, R. , Clissold, L. , Baker, D. *et al* (2011) Dissecting the genome of the polyploid crop oilseed rape by transcriptome sequencing. Nat. Biotechnol. 29, 762–766. 21804563 10.1038/nbt.1926

[pbi13262-bib-0006] Bayer, P.E. , Hurgobin, B. , Golicz, A.A. , Chan, C.K.K. , Yuan, Y. , Lee, H.T. , Renton, M. *et al* (2017) Assembly and comparison of two closely related *Brassica napus* genomes. Plant Biotechnol. J. 15, 1602–1610. 28403535 10.1111/pbi.12742 PMC5698052

[pbi13262-bib-0007] Bayer, P.E. , Golicz, A.A. , Tirnaz, S. , Chan, C.K.K. , Edwards, D. and Batley, J. (2018) Variation in abundance of predicted resistance genes in the *Brassica oleracea* pangenome. Plant Biotechnol. J. 17, 789–800. 30230187 10.1111/pbi.13015 PMC6419861

[pbi13262-bib-0008] Cai, C. , Wang, X. , Liu, B. , Liang, J. , Cui, Y. , Cheng, F. and Wang, X. (2017) *Brassica rapa* Genome 2.0: a reference upgrade through sequence re‐assembly and gene re‐annotation. Mol. Plant Cell Press, 10, 649–651. 10.1016/j.molp.2016.11.008 27890636

[pbi13262-bib-0009] Chalhoub, B. , Denoeud, F. , Liu, S. , Parkin, I.A.P. , Tang, H. , Wang, X. , Chiquet, J. *et al* (2014) Early allopolyploid evolution in the post‐Neolithic *Brassica napus* oilseed genome. Science, 345, 950–953. 25146293 10.1126/science.1253435

[pbi13262-bib-0010] Chen, J.Y. , Huang, J.Q. , Li, N.Y. , Ma, X.F. , Wang, J.L. , Liu, C. , Liu, Y.F. *et al* (2015) Genome‐wide analysis of the gene families of resistance gene analogues in cotton and their response to Verticillium Wilt. BMC Plant Biol. 15, 148. 26084488 10.1186/s12870-015-0508-3 PMC4471920

[pbi13262-bib-0012] Cingolani, P. , Platts, A. , le Wang, L. , Coon, M. , Nguyen, T. , Wang, L. , Land, S.J. *et al* (2012) A program for annotating and predicting the effects of single nucleotide polymorphisms, SnpEff: SNPs in the genome of *Drosophila melanogaster* strain w1118; iso‐2; iso‐3. Fly (Austin), 6, 80–92. 22728672 10.4161/fly.19695 PMC3679285

[pbi13262-bib-0013] Danecek, P. , Auton, A. , Abecasis, G. , Albers, C.A. , Banks, E. , DePristo, M.A. , Handsaker, R.E. *et al* (2011) The variant call format and VCFtools. Bioinformatics, 27, 2156–2158. 21653522 10.1093/bioinformatics/btr330 PMC3137218

[pbi13262-bib-0014] Dangl, J.L. and Jones, J.D. (2001) Plant pathogens and integrated defence responses to infection. Nature, 411, 826–833. 11459065 10.1038/35081161

[pbi13262-bib-0015] Delourme, R. , Pilet‐Nayel, M.L. , Archipiano, M. , Horvais, R. , Tanguy, X. , Rouxel, T. , Brun, H. *et al* (2004) A cluster of major specific resistance genes to *Leptosphaeria maculans* in *Brassica napus* . Phytopathology, 94, 578–583. 18943482 10.1094/PHYTO.2004.94.6.578

[pbi13262-bib-0016] Edwards, D. , Batley, J. , Parkin, I. and Kole, C. (2011) Genetics, Genomics and Breeding of Oilseed Brassicas. Boca Raton, FL: CRC Press, 13 Sep Science 438 pages.

[pbi13262-bib-0017] Feltus, F.A. , Wan, J. , Schulze, S.R. , Estill, J.C. , Jiang, N. and Paterson, A.H. (2004) An SNP resource for rice genetics and breeding based on subspecies indica and japonica genome alignments. Genome Res. 14, 1812–1819. 15342564 10.1101/gr.2479404 PMC515328

[pbi13262-bib-0018] Friedman, A.R. and Baker, B.J. (2007) The evolution of resistance genes in multi‐protein plant resistance systems. Curr. Opin. Genet. Develop. 17, 493–499. 10.1016/j.gde.2007.08.014 17942300

[pbi13262-bib-0019] Gaeta, R.T. , Pires, J.C. , Iniguez‐Luy, F. , Leon, E. and Osborn, T.C. (2007) Genomic changes in resynthesized *Brassica napus* and their effect on gene expression and phenotype. Plant Cell, 19, 3403–3417. 18024568 10.1105/tpc.107.054346 PMC2174891

[pbi13262-bib-0020] Gan, X. , Stegle, O. , Behr, J. , Steffen, J.G. , Drewe, P. , Hildebrand, K.L. , Lyngsoe, R. *et al* (2011) Multiple reference genomes and transcriptomes for *Arabidopsis thaliana* . Nature, 477, 419–423. 21874022 10.1038/nature10414 PMC4856438

[pbi13262-bib-0021] Gel, B. and Serra, E. (2017) karyoploteR: an R/Bioconductor package to plot customizable genomes displaying arbitrary data. Bioinformatics, 1(), 3088–3090. 10.1093/bioinformatics/btx346 PMC5870550 28575171

[pbi13262-bib-0022] Golicz, A.A. , Martinez, P.A. , Zander, M. , Patel, D.A. , Van De Wouw, A.P. , Visendi, P. , Fitzgerald, T.L. *et al* (2015) Gene loss in the fungal canola pathogen *Leptosphaeria maculans* . Funct. Integr. Genomics, 15, 189–196. 25421464 10.1007/s10142-014-0412-1

[pbi13262-bib-0023] Golicz, A.A. , Bayer, P.E. , Barker, G.C. , Edger, P.P. , Kim, H. , Martinez, P.A. , Chan, C.K.K. *et al* (2016a) The pangenome of an agronomically important crop plant *Brassica oleracea* . Nat. Commun. 7, 13390. 27834372 10.1038/ncomms13390 PMC5114598

[pbi13262-bib-0024] Golicz, A.A. , Batley, J. and Edwards, D. (2016b) Towards plant pangenomics. Plant Biotechnol. J. 4, 1099–1105. 10.1111/pbi.12499 PMC11388911 26593040

[pbi13262-bib-0025] Guo, Y.L. , Fitz, J. , Schneeberger, K. , Ossowski, S. , Cao, J. and Weigel, D. (2011) Genome‐wide comparison of nucleotide‐binding site‐leucine‐rich repeat‐encoding genes in *Arabidopsis* . Plant Physiol. 157, 757–769. 21810963 10.1104/pp.111.181990 PMC3192553

[pbi13262-bib-0026] Hammond‐Kosack, K.E. and Jones, J.D. (1997) Plant disease resistance genes. Annu. Rev. Plant Physiol. Plant Mol. Biol. 48, 575–607. 15012275 10.1146/annurev.arplant.48.1.575

[pbi13262-bib-0027] Hirsch, C.N. , Foerster, J.M. , Johnson, J.M. , Sekhon, R.S. , Muttoni, G. , Vaillancourt, B. , Peñagaricano, F. *et al* (2014) Insights into the maize pan‐genome and pan‐transcriptome. Plant Cell, 26, 121–135. 24488960 10.1105/tpc.113.119982 PMC3963563

[pbi13262-bib-0028] Holliday, R. and Grigg, G.W. (1993) DNA methylation and mutation. Mutat. Res. 285, 61–67. 7678134 10.1016/0027-5107(93)90052-h

[pbi13262-bib-0029] Holub, E.B. (2001) The arms race is ancient history in *Arabidopsis*, the wildflower. Nat. Rev. Genet. 2, 516–527. 11433358 10.1038/35080508

[pbi13262-bib-0030] Huang, S. , Deng, L. , Guan, M. , Li, J. , Lu, K. , Wang, H. , Fu, D. *et al* (2013) Identification of genome‐wide single nucleotide polymorphisms in allopolyploid crop *Brassica napus* . BMC Genom. 14, 717. 10.1186/1471-2164-14-717 PMC4046652 24138473

[pbi13262-bib-0031] Huang, Y.J. , Jestin, C. , Welham, S.J. , King, G.J. , Manzanares‐Dauleux, M.J. , Fitt, B.D.L. and Delourme, R. (2016) Identification of environmentally stable QTL for resistance against *Leptosphaeria maculans* in oilseed rape (*Brassica napus*). Theoret. Appl. Genet. 129, 169–180. 26518572 10.1007/s00122-015-2620-z PMC4703627

[pbi13262-bib-0032] Hulbert, S.H. , Webb, C.A. , Smith, S.M. and Sun, Q. (2001) Resistance gene complexes: evolution and utilization. Annu. Rev. Phytopathol. 39, 285–312. 11701867 10.1146/annurev.phyto.39.1.285

[pbi13262-bib-0033] Hurgobin, B. and Edwards, D. (2017) SNP discovery using a pangenome: has the single reference approach become obsolete? Biology, 6, 21. 10.3390/biology6010021 PMC5372014 28287462

[pbi13262-bib-0034] Hurgobin, B. , Golicz, A.A. , Bayer, P.E. , Chan, C.K.K. , Tirnaz, S. , Dolatabadian, A. , Schiessl, S.V. *et al* (2018) Homoeologous exchange is a major cause of gene presence/absence variation in the amphidiploid *Brassica napus* . Plant Biotechnol. J. 16, 1265–1274. 29205771 10.1111/pbi.12867 PMC5999312

[pbi13262-bib-0035] Jeong, I.S. , Yoon, U.H. , Lee, G.S. , Ji, H.S. , Lee, H.J. , Han, C.D. , Hahn, J.H. *et al* (2013) SNP‐based analysis of genetic diversity in anther‐derived rice by whole genome sequencing. Rice, 14(), 6. 10.1186/1939-8433-6-6 PMC4883692 24280451

[pbi13262-bib-0036] Jestin, C. , Lodé, M. , Vallée, P. , Domin, C. , Falentin, C. , Horvais, R. , Coedel, S. *et al* (2011) Association mapping of quantitative resistance for *Leptosphaeria maculans* in oilseed rape (*Brassica napus* L.). Mol. Breed. 27, 271–287.

[pbi13262-bib-0037] Kale, S.M. , Pardeshi, V.C. , Barvkar, V.T. , Gupta, V.S. and Kadoo, N.Y. (2013) Genome‐wide identification and characterization of nucleotide binding site leucine‐rich repeat genes in linseed reveal distinct patterns of gene structure. Genome, 56, 91–99. 23517318 10.1139/gen-2012-0135

[pbi13262-bib-0038] Kohler, A. , Rinaldi, C. , Duplessis, S. , Baucher, M. , Geelen, D. , Duchaussoy, F. , Meyers, B.C. *et al* (2008) Genome‐wide identification of NBS resistance genes in *Populus trichocarpa* . Plant Mol. Biol. 66, 619–636. 18247136 10.1007/s11103-008-9293-9

[pbi13262-bib-0039] Kruijt, M. , DE Kock, M.J. and de Wit, P.J. . (2005) Receptor‐like proteins involved in plant disease resistance. Mol. Plant Pathol. 6, 85–97. 20565641 10.1111/j.1364-3703.2004.00264.x

[pbi13262-bib-0040] Kuang, H. , Woo, S.S. , Meyers, B.C. , Nevo, E. and Michelmore, R.W. (2004) Multiple genetic processes result in heterogeneous rates of evolution within the major cluster disease resistance genes in lettuce. Plant Cell, 16, 2870–2894. 15494555 10.1105/tpc.104.025502 PMC527186

[pbi13262-bib-0041] Larkan, N.J. , Raman, H. , Lydiate, D.J. , Robinson, S.J. , Yu, F. , Barbulescu, D.M. , Raman, R. *et al* (2016) Multi‐environment QTL studies suggest a role for cysteine‐rich protein kinase genes in quantitative resistance to blackleg disease in *Brassica napus* . BMC Plant Biol. 16, 183. 27553246 10.1186/s12870-016-0877-2 PMC4995785

[pbi13262-bib-0042] Leflon, M. , Brun, H. , Eber, F. , Delourme, R. , Lucas, M.O. , Vallée, P. , Ermel, M. *et al* (2007) Detection, introgression and localization of genes conferring specific resistance to *Leptosphaeria maculans* from *Brassica rapa* into *B. napus* . Theoret. Appl. Genet. 115, 897–906. 17668174 10.1007/s00122-007-0616-z

[pbi13262-bib-0043] Leister, D. (2004) Tandem and segmental gene duplication and recombination in the evolution of plant disease resistance gene. Trends Genet. 20, 116–122. 15049302 10.1016/j.tig.2004.01.007

[pbi13262-bib-0044] Li, Y.H. , Zhou, G. , Ma, J. , Jiang, W. , Jin, L.G. , Zhang, Z. , Guo, Y. *et al* (2014) De novo assembly of soybean wild relatives for pan‐genome analysis of diversity and agronomic traits. Nat. Biotechnol. 32, 1045–1052. 25218520 10.1038/nbt.2979

[pbi13262-bib-0045] Li, P. , Quan, X. , Jia, G. , Xiao, J. , Cloutier, S. and You, F.M. (2016) RGAugury: a pipeline for genome‐wide prediction of resistance gene analogs (RGAs) in plants. BMC Genom. 17, 852. 10.1186/s12864-016-3197-x PMC5093994 27806688

[pbi13262-bib-0046] Li, Y. , Wei, W. , Feng, J. , Luo, H. , Pi, M. , Liu, Z. and Kang, C. (2017) Genome re‐annotation of the wild strawberry Fragaria vesca using extensive Illumina‐and SMRT‐based RNA‐seq datasets. DNA Res. 25, 61–70. 10.1093/dnares/dsx038 PMC5824900 29036429

[pbi13262-bib-0047] Lin, K. , Zhang, N. , Severing, E.I. , Nijveen, H. , Cheng, F. , Visser, R.G. , Wang, X. *et al* (2014) Beyond genomic variation–comparison and functional annotation of three *Brassica rapa* genomes: a turnip, a rapid cycling and a Chinese cabbage. BMC Genom. 15, 250. 10.1186/1471-2164-15-250 PMC4230417 24684742

[pbi13262-bib-0048] Liu, S. , Liu, Y. , Yang, X. , Tong, C. , Edwards, D. , Parkin, I.A.P. , Zhao, M. *et al* (2014) The *Brassica oleracea* genome reveals the asymmetrical evolution of polyploid genomes. Nat. Commun. 5, 3930. 24852848 10.1038/ncomms4930 PMC4279128

[pbi13262-bib-0049] Marchal, C. , Zhang, J. , Zhang, P. , Fenwick, P. , Steuernagel, B. , Adamski, N.M. , Boyd, L. *et al* (2018) BED‐domain containing immune receptors confer diverse resistance spectra to yellow rust. Nature Plants, 4, 662–668. 30150615 10.1038/s41477-018-0236-4

[pbi13262-bib-0050] McLaren, W. , Gil, L. , Hunt, S.E. , Riat, H.S. , Ritchie, G.R. , Thormann, A. , Flicek, P. *et al* (2016) The ensembl variant effect predictor. Genome Biol. 17, 122. 27268795 10.1186/s13059-016-0974-4 PMC4893825

[pbi13262-bib-0051] Meyers, B. , Kozik, A. , Griego, A. , Kuang, H. and Michelmore, R. (2003) Genome‐wide analysis of NBS‐LRR‐encoding genes in *Arabidopsis* . Plant Cell, 15, 809–834. 12671079 10.1105/tpc.009308 PMC152331

[pbi13262-bib-0052] Montenegro, J.D. , Golicz, A.A. , Bayer, P.E. , Hurgobin, B. , Lee, H. , Chan, C.K.K. , Visendi, P. *et al* (2017) The pangenome of hexaploid bread wheat. Plant J. 90, 1007–1013. 28231383 10.1111/tpj.13515

[pbi13262-bib-0053] Morris, E.R. and Walker, J.C. (2003) Receptor‐like protein kinases: the keys to response. Curr. Opin. Plant Biol. 6, 339–342. 12873528 10.1016/s1369-5266(03)00055-4

[pbi13262-bib-0054] Mun, J.H. , Yu, H.J. , Park, S. and Park, B.S. (2009) Genome‐wide identification of NBS‐encoding resistance genes in *Brassica rapa* . Mol. Genet. Genom. 282, 617–631. 10.1007/s00438-009-0492-0 PMC2777221 19838736

[pbi13262-bib-0055] Parkin, I. , Koh, C. , Tang, H. , Robinson, S. , Kagale, S. , Clarke, W. , Town, C. *et al* (2014) Transcriptome and methylome profiling reveals relics of genome dominance in the mesopolyploid *Brassica oleracea* . Genome Biol. 15, R77. 24916971 10.1186/gb-2014-15-6-r77 PMC4097860

[pbi13262-bib-0056] Pootakham, W. , Jomchai, N. , Ruang‐Areerate, P. , Shearman, J.R. , Sonthirod, C. , Sangsrakru, D. , Tragoonrung, S. *et al* (2015) Genome‐wide SNP discovery and identification of QTL associated with agronomic traits in oil palm using genotyping‐by‐sequencing (GBS). Genomics, 105, 288–295. 25702931 10.1016/j.ygeno.2015.02.002

[pbi13262-bib-0057] Porter, B.W. , Paidi, M. , Ming, R. , Alam, M. , Nishijima, W.T. and Zhu, Y.J. (2009) Genome‐wide analysis of Carica papaya reveals a small NBS resistance gene family. Mol. Genet. Genom. 281, 609–626. 10.1007/s00438-009-0434-x 19263082

[pbi13262-bib-0058] Qiu, L.J. , Xing, L.L. , Guo, Y. , Wang, J. , Jackson, S.A. and Chang, R.Z. (2013) A platform for soybean molecular breeding: the utilization of core collections for food security. Plant Mol. Biol. 83, 41–50. 23708950 10.1007/s11103-013-0076-6 PMC3755216

[pbi13262-bib-0059] R Core Team (2016) R: A Language and Environment for Statistical Computing. Vienna: R Foundation for Statistical Computing.

[pbi13262-bib-0060] Raman, R. , Taylor, B. , Lindbeck, K. , Coombes, N. , Barbulescu, D. , Salisbury, P. and Raman, H. (2012a) Molecular mapping and validation of *Rlm1* gene for resistance to *Leptosphaeria maculans* in canola (*Brassica napus* L.). Crop Pasture Sci. 63, 1007–1017.

[pbi13262-bib-0061] Raman, R. , Taylor, B. , Marcroft, S. , Stiller, J. , Eckermann, P. , Coombes, N. , Rehman, A. *et al* (2012b) Molecular mapping of qualitative and quantitative loci for resistance to *Leptosphaeria maculans* causing blackleg disease in canola (*Brassica napus* L.). Theoret. Appl. Genet. 125, 405–418. 22454144 10.1007/s00122-012-1842-6

[pbi13262-bib-0062] Rice Chromosomes 11 and 12 Sequencing Consortia (2005) The sequence of rice chromosomes 11 and 12, rich in disease resistance genes and recent gene duplications. BMC Biol. 3, 20. 16188032 10.1186/1741-7007-3-20 PMC1261165

[pbi13262-bib-0063] Richly, E. , Kurth, J. and Leister, D. (2002) Mode of amplification and reorganization of resistance genes during recent *Arabidopsis thaliana* evolution. J. Mol. Evol. 19, 76–84. 10.1093/oxfordjournals.molbev.a003984 11752192

[pbi13262-bib-0064] Saxena, R.K. , Edwards, D. and Varshney, R.K. (2014) Structural variations in plant genomes. Brief Funct. Genom. 13, 296–307. 10.1093/bfgp/elu016 PMC4110416 24907366

[pbi13262-bib-0065] Sekhwal, M.K. , Li, P. , Lam, I. , Wang, X. , Cloutier, S. and You, F.M. (2015) Disease resistance gene analogs (RGAs) in plants. Int. J. Mol. Sci. 16, 19248–19290. 26287177 10.3390/ijms160819248 PMC4581296

[pbi13262-bib-0066] Shao, Z.Q. , Xue, J.Y. , Wu, P. , Zhang, Y.M. , Wu, Y. , Hang, Y.Y. , Wang, B. *et al* (2016) Large‐scale analyses of angiosperm nucleotide‐binding site‐leucine‐rich repeat genes reveal three anciently diverged classes with distinct evolutionary patterns. Plant Physiol. 170, 2095–2109. 26839128 10.1104/pp.15.01487 PMC4825152

[pbi13262-bib-0067] Shao, Z.Q. , Xue, J.Y. , Wang, Q. , Wang, B. and Chen, J.Q. (2019) Revisiting the origin of plant NBS‐LRR genes. Trends Plant Sci. 24, 9–12. 30446304 10.1016/j.tplants.2018.10.015

[pbi13262-bib-0068] Singh, S. , Chand, S. , Singh, N.K. and Sharma, T.R. (2015) Genome‐wide distribution, organisation and functional characterization of disease resistance and defence response genes across rice species. PLoS ONE, 10(), e0125964. 25902056 10.1371/journal.pone.0125964 PMC4406684

[pbi13262-bib-0069] Skidmore, Z.L. , Wagner, A.H. , Lesurf, R. , Campbell, K.M. , Kunisaki, J. , Griffith, O.L. and Griffith, M. (2016) GenVisR: genomic visualizations in R. Bioinformatics, 32, 3012–3014. 27288499 10.1093/bioinformatics/btw325 PMC5039916

[pbi13262-bib-0070] Smith, S.M. , Pryor, A.J. and Hulbert, S.H. (2004) Allelic and haplotypic diversity at the rp1 rust resistance locus of maize. Genetics, 167, 1939–1947. 15342531 10.1534/genetics.104.029371 PMC1471013

[pbi13262-bib-0071] Song, K.M. , Lu, P. , Tang, K.L. and Osborn, T.C. (1995) Rapid genome change in synthetic polyploids of *Brassica* and its implications for polyploid evolution. Proc. Natl Acad. Sci. USA, 92, 7719–7723. 7644483 10.1073/pnas.92.17.7719 PMC41217

[pbi13262-bib-0072] Subbaiyan, G.K. , Waters, D.L.E. , Katiyar, S.K. , Sadananda, A.R. , Vaddadi, S. and Henry, R.J. (2012) Genome‐wide DNA polymorphisms in elite indica rice inbreds discovered by whole‐genome sequencing. Plant Biotechnol. J. 10, 623–634. 22222031 10.1111/j.1467-7652.2011.00676.x

[pbi13262-bib-0073] Sun, Q. , Li, Lin , Liu, D. , Wu, D. , Fang, Y. , Wu, J. and Wang, Y. (2018) CRISPR/Cas9‐mediated multiplex genome editing of the BnWRKY11 and BnWRKY70 genes in *Brassica napus* L. Int. J. Mol. Sci. 19, 2716. 10.3390/ijms19092716 PMC6163266 30208656

[pbi13262-bib-0074] Szadkowski, E. , Eber, F. , Huteau, V. , Lodé, M. , Huneau, C. , Belcram, H. , Coriton, O. *et al* (2010) The first meiosis of resynthesized Brassica napus, a genome blender. New Phytol. 186, 102–112. 20149113 10.1111/j.1469-8137.2010.03182.x

[pbi13262-bib-0075] Tang, S. and Zhao, J. (2015) Patent Application Publication, Pub No US 2015/0074851 A1.

[pbi13262-bib-0076] Tollenaere, R. , Hayward, A. , Dalton‐Morgan, J. , Campbell, E. , Lee, J.R. , Lorenc, M.T. , Manoli, S. *et al* (2012) Identification and characterization of candidate *Rlm4* blackleg resistance genes in *Brassica napus* using next‐generation sequencing. Plant Biotechnol. J. 10, 709–715. 22726421 10.1111/j.1467-7652.2012.00716.x

[pbi13262-bib-0077] UN (1935) Genome analysis in Brassica with special reference to the experimental formation of *B. napus* and peculiar mode of fertilization. Japanese J. Botany, 7, 389–452.

[pbi13262-bib-0078] Ve, T. , Williams, S.J. and Kobe, B. (2015) Structure and function of Toll/interleukin‐1 receptor/resistance protein (TIR) domains. Apoptosis, 20, 250–261. 25451009 10.1007/s10495-014-1064-2

[pbi13262-bib-0079] van der Vossen, E.A. , van der Voort, J.N. , Kanyuka, K. , Bendahmane, A. , Sandbrink, H. , Baulcombe, D.C. , Bakker, J. *et al* (2000) Homologues of a single resistance‐gene cluster in potato confer resistance to distinct pathogens: a virus and a nematode. Plant J. 23, 567–576. 10972883 10.1046/j.1365-313x.2000.00814.x

[pbi13262-bib-0080] Wang, X. , Wang, H. , Wang, J. , Sun, R. , Wu, J. , Liu, S. , Bai, Y. *et al* (2011) The genome of the mesopolyploid crop species *Brassica rapa* . Nat. Genet. 43, 1035–1039. 21873998 10.1038/ng.919

[pbi13262-bib-0081] Wondji, C.S. , Hemingway, J. and Ranson, H. (2007) Identification and analysis of Single Nucleotide Polymorphisms (SNPs) in the mosquito *Anopheles funestus*, malaria vector. BMC Genom. 8, 5. 10.1186/1471-2164-8-5 PMC1781065 17204152

[pbi13262-bib-0082] Wu, P. , Shao, Z.Q. , Wu, X.Z. , Wang, Q. , Wang, B. , Chen, J.Q. , Hang, Y.Y. *et al* (2014) Loss/retention and evolution of NBS‐encoding genes upon whole genome triplication of *Brassica rapa* . Gene, 540, 54–61. 24576745 10.1016/j.gene.2014.01.082

[pbi13262-bib-0083] Xiong, Z. , Gaeta, R.T. and Pires, C. (2011) Homoeologous shuffling and chromosome compensation maintain genome balance in resynthesized allopolyploid *Brassica napus* . Proc. Natl Acad. Sci. USA, 108, 7908–7913. 21512129 10.1073/pnas.1014138108 PMC3093481

[pbi13262-bib-0084] Yang, S. , Zhang, X. , Yue, J.X. , Tian, D. and Chen, J.Q. (2008) Recent duplications dominate NBS‐encoding gene expansion in two woody species. Mol. Genet. Genom. 280, 187–198. 10.1007/s00438-008-0355-0 18563445

[pbi13262-bib-0085] Yang, J. , Liu, D. , Wang, X. , Ji, C. , Cheng, F. , Liu, B. , Hu, Z. *et al* (2016) The genome sequence of allopolyploid *Brassica juncea* and analysis of differential homoeolog gene expression influencing selection. Nat. Genet. 48, 1225–1234. 27595476 10.1038/ng.3657

[pbi13262-bib-0086] Yao, W. , Li, G. , Zhao, H. , Wang, G. , Lian, X. and Xi, W. (2015) Exploring the rice dispensable genome using a metagenome‐like assembly strategy. Genome Biol. 16, 1–20. 26403182 10.1186/s13059-015-0757-3 PMC4583175

[pbi13262-bib-0087] Yu, J. , Tehrim, S. , Zhang, F.Q. , Tong, C.B. , Huang, J.Y. , Cheng, X.H. , Dong, C.H. *et al* (2014) Genome‐wide comparative analysis of NBS‐encoding genes between *Brassica* species and *Arabidopsis thaliana* . BMC Genom. 15, 3. 10.1186/1471-2164-15-3 PMC4008172 24383931

[pbi13262-bib-0088] Zheng, L.Y. , Guo, X.S. , He, B. , Sun, L.J. , Peng, Y. , Dong, S.S. , Liu, T.F. *et al* (2011) Genome‐wide patterns of genetic variation in sweet and grain sorghum (*Sorghum bicolor*). Genome Biol. 12, R114. 22104744 10.1186/gb-2011-12-11-r114 PMC3334600

[pbi13262-bib-0089] Zhou, T. , Wang, Y. , Chen, J.Q. , Araki, H. , Jing, Z. , Jiang, K. , Shen, J. *et al* (2004) Genome‐wide identification of NBS genes in japonica rice reveals significant expansion of divergent non‐TIR NBS‐LRR genes. Mol. Genet. Genom. 271, 402–415. 10.1007/s00438-004-0990-z 15014983

[pbi13262-bib-0090] Zhu, H. , Cannon, S.B. , Young, N.D. and Cook, D.R. (2002) Phylogeny and genomic organization of the TIR and Non‐TIR NBS‐LRR resistance gene family in *Medicago truncatula* . Mol. Plant Microbe Interact. 15, 529–539. 12059101 10.1094/MPMI.2002.15.6.529

[pbi13262-bib-0091] Zhu, B. , Xiang, Y. , Zeng, P. , Cai, B. , Huang, X. , Ge, X. , Weng, Q. *et al* (2018) Genome‐wide gene expression disturbance by single A1/C1 chromosome substitution in *Brassica rapa* restituted from natural *B. napus* . Frontiers Plant Sci. 9, 377. 10.3389/fpls.2018.00377 PMC5870043 29616075

