# Supplementary material for: Characterization of disease resistance genes in the Brassica napus pangenome reveals significant structural variation
Source: Plant Biotechnol J. 2019 Oct 10;18(4):969–82. doi: 10.1111/pbi.13262 (PMC7061875; doi:10.1111/pbi.13262)
Supplement: Supplementary file 1 — Figure S1 The absolute number of RGAs on the reference genomes. Figure S2 The distribution of stop codons (stop_gained, stop_lost and stop_retained_variant) across the 50 non‐synthetic (fodder (blue), swede (red), vegetable (green) and oilseed (grey)) and synthetic (black) lines on the reference genome, pangenome additional contigs and reference genome unplaced contigs. Figure S3 Physical clustering of NBS‐LRR genes on the chromosome of the A genome of B. napus. The colourful circles above (variable) and below (core) each chromosome (grey bars) are designated for NBS classes. Chromosome lengths are shown in megabase pairs on the scale at the top. Figure S4 Physical clustering of NBS‐LRR genes on the chromosome of the C genome of B. napus. The colourful circles above (variable) and below (core) each chromosome (grey bars) are designated for NBS classes. Chromosome lengths are shown in megabase pairs on the scale at the top. Figure S5 Physical clustering of TM‐LRR genes on the chromosome of the A genome of B. napus. The colourful circles above (variable) and below (core) each chromosome (grey bars) are designated for NBS classes. Chromosome lengths are shown in megabase pairs on the scale at the top. Figure S6 Physical clustering of TM‐LRR genes on the chromosome of the C genome of B. napus. The colourful circles above (variable) and below (core) each chromosome (grey bars) are designated for NBS classes. Chromosome lengths are shown in megabase pairs on the scale at the top. Figure S7 Rlm1, Rlm3, Rlm4, Rlm7 and Rlm9 QTL were combined into non‐redundant QTL regions by combining QTL length overlaps into single contiguous regions. Non‐QTL regions contain no QTL whatsoever. [file PBI-18-969-s003.pdf]

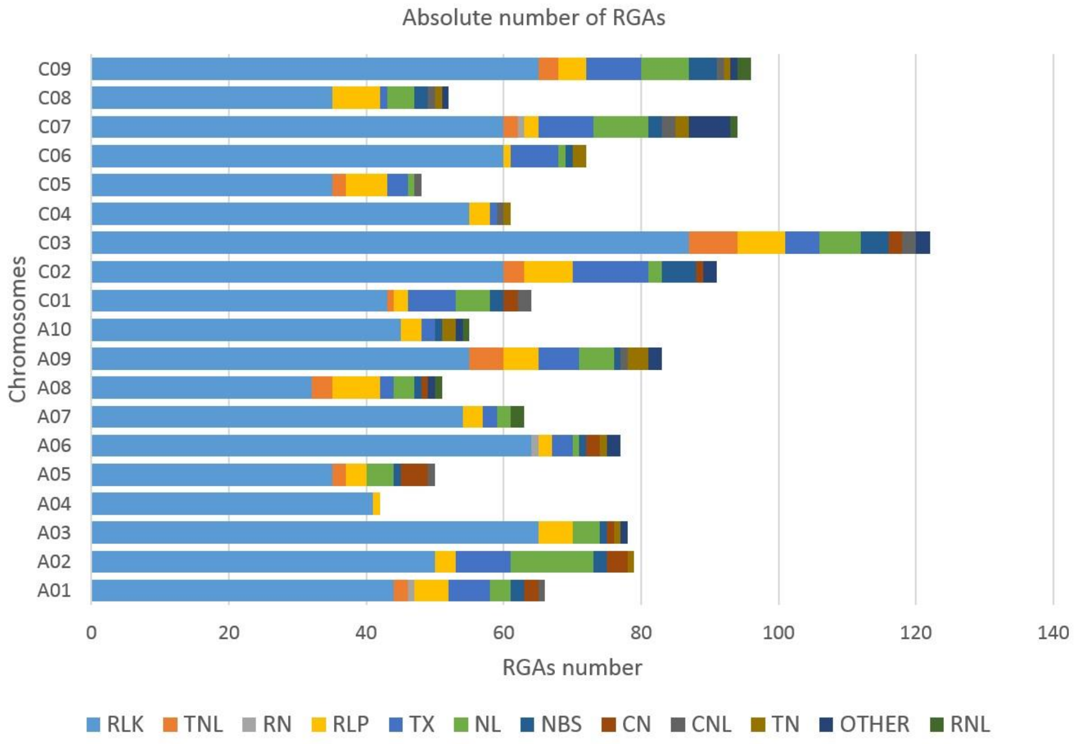

Supplementary Figure 1: The absolute number of RGAs on the reference genomes

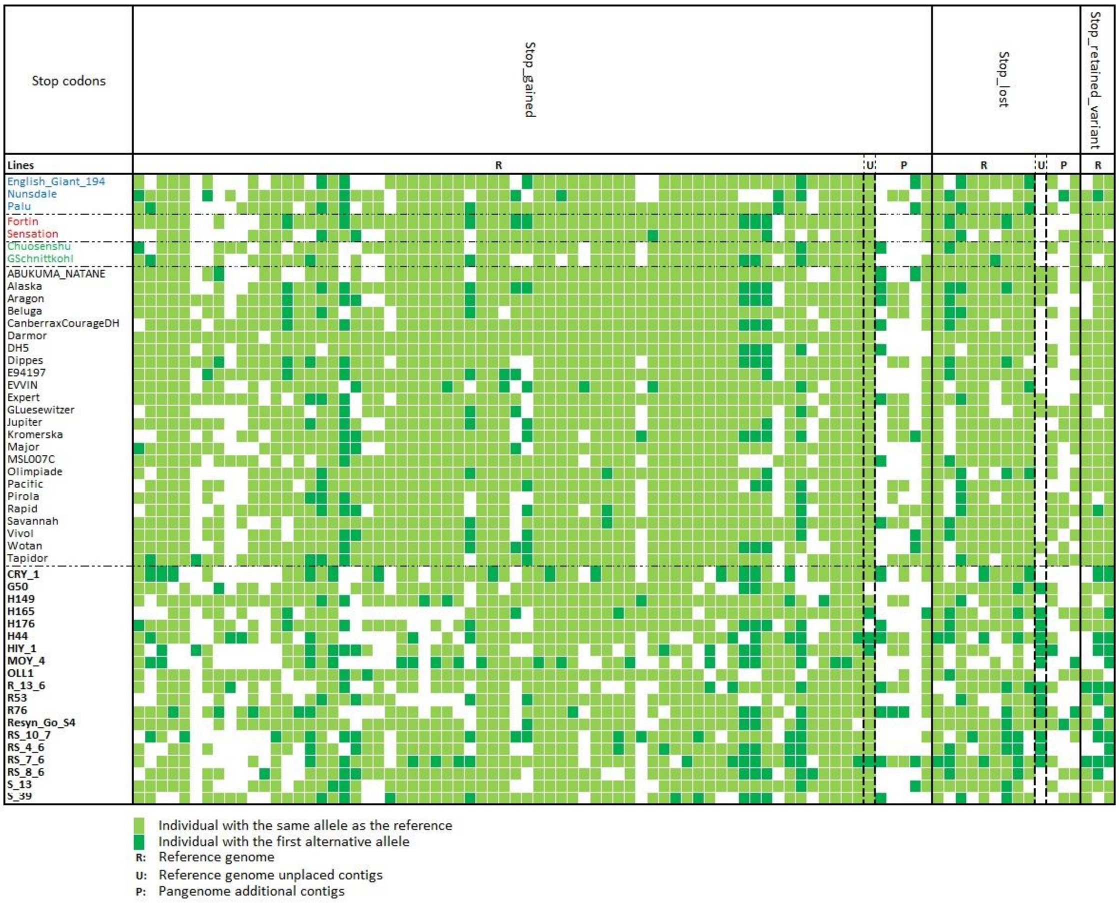

Supplementary Figure 2: The distribution of stop codons (stop_gained, stop_lost and stop_retained_variant) across the 50 non-synthetic (fodder (blue), swede (red), vegetable (green) and oilseed (grey)) and synthetic (black) lines on the reference genome (R), pangneome additional contigs (P) and reference genome unplaced contigs (U).

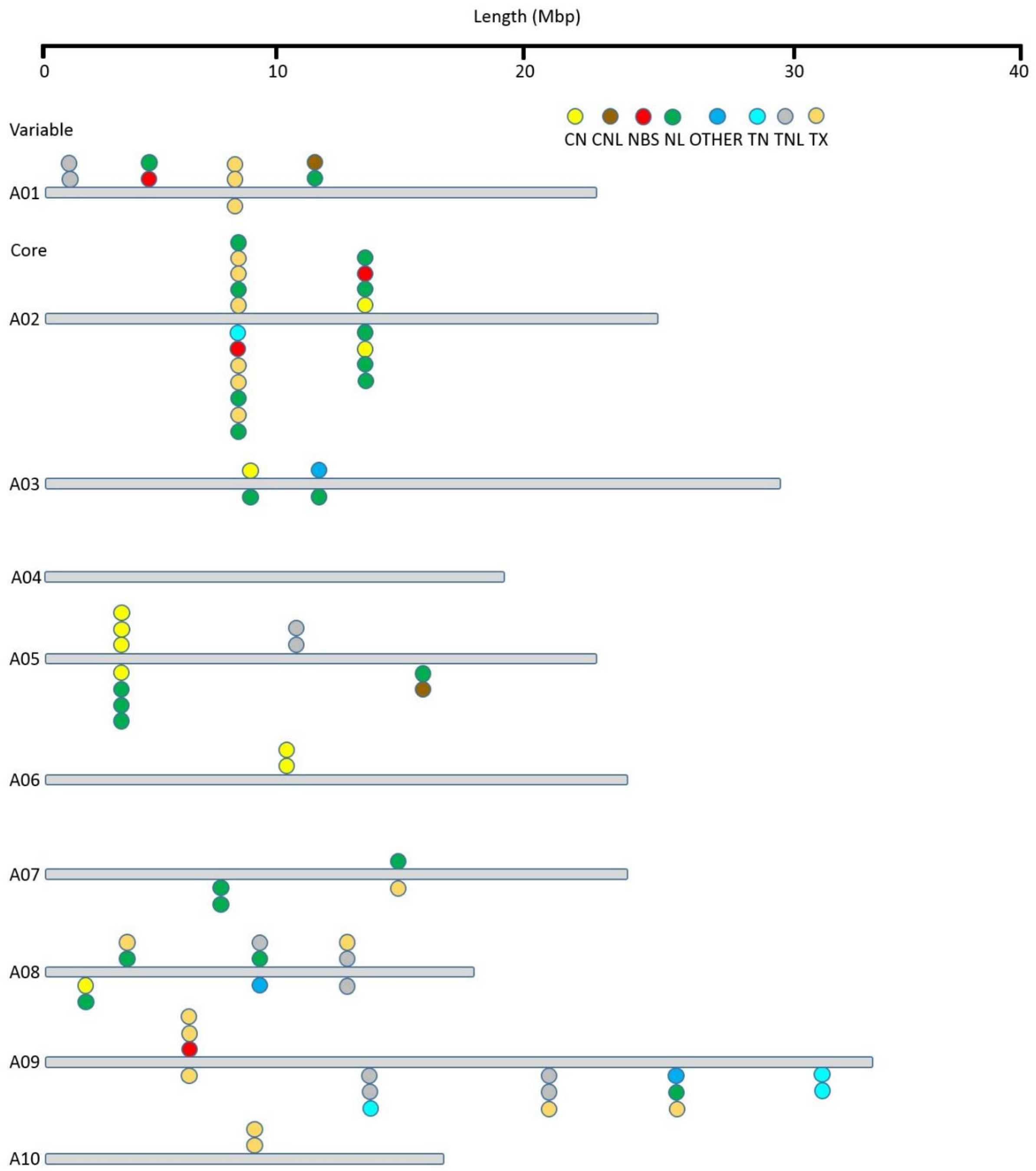

Supplementary Figure 3: Physical clustering of NBS-LRR genes on the chromosome of the A genome of *B. napus*. The colourful circles above (variable) and below (core) each chromosome (grey bars) are designated for NBS classes. Chromosome lengths are shown in megabase pairs on the scale at the top.

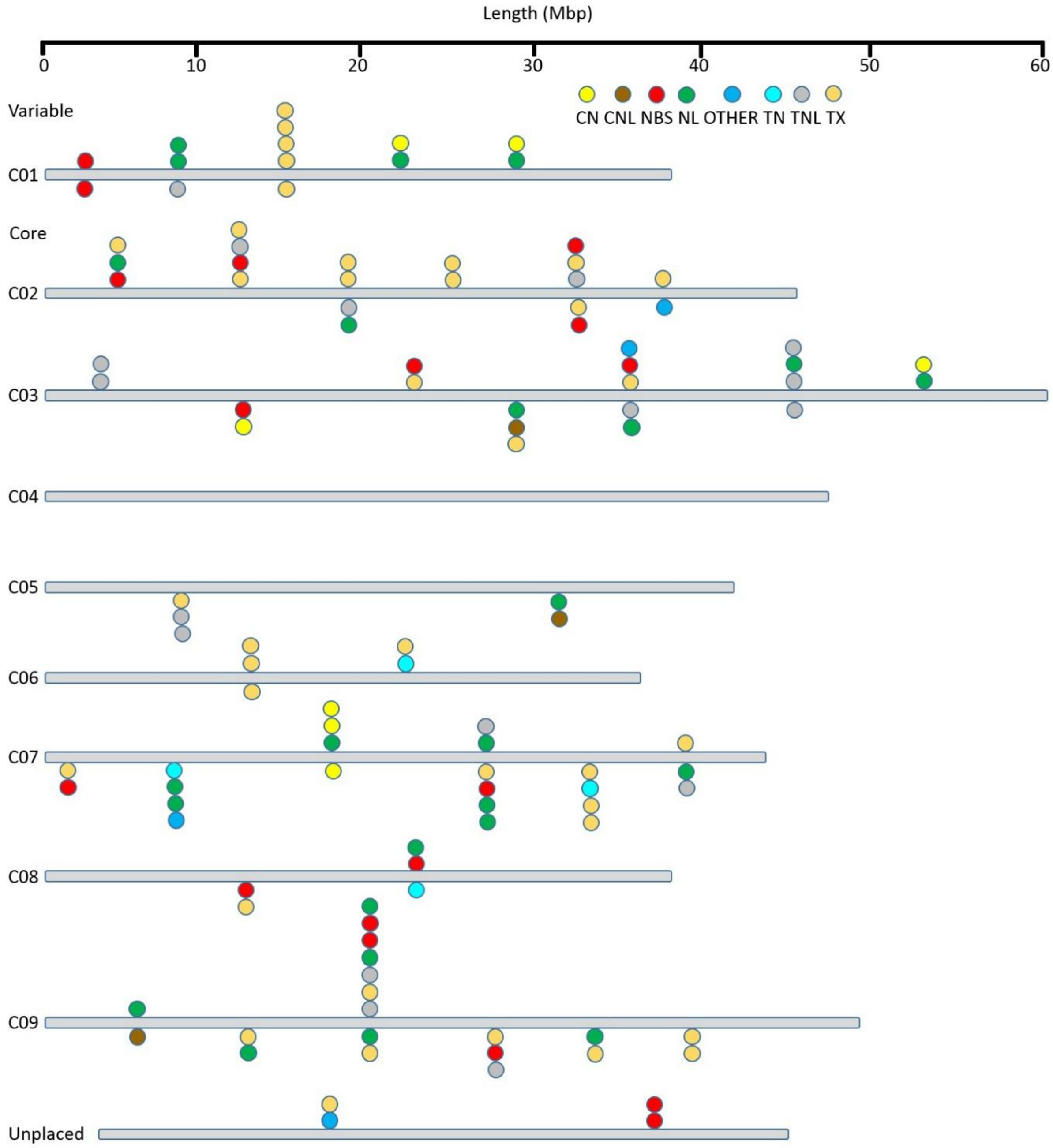

Supplementary Figure 4: Physical clustering of NBS-LRR genes on the chromosome of the C genome of *B. napus*. The colourful circles above (variable) and below (core) each chromosome (grey bars) are designated for NBS classes. Chromosome lengths are shown in megabase pairs on the scale at the top.

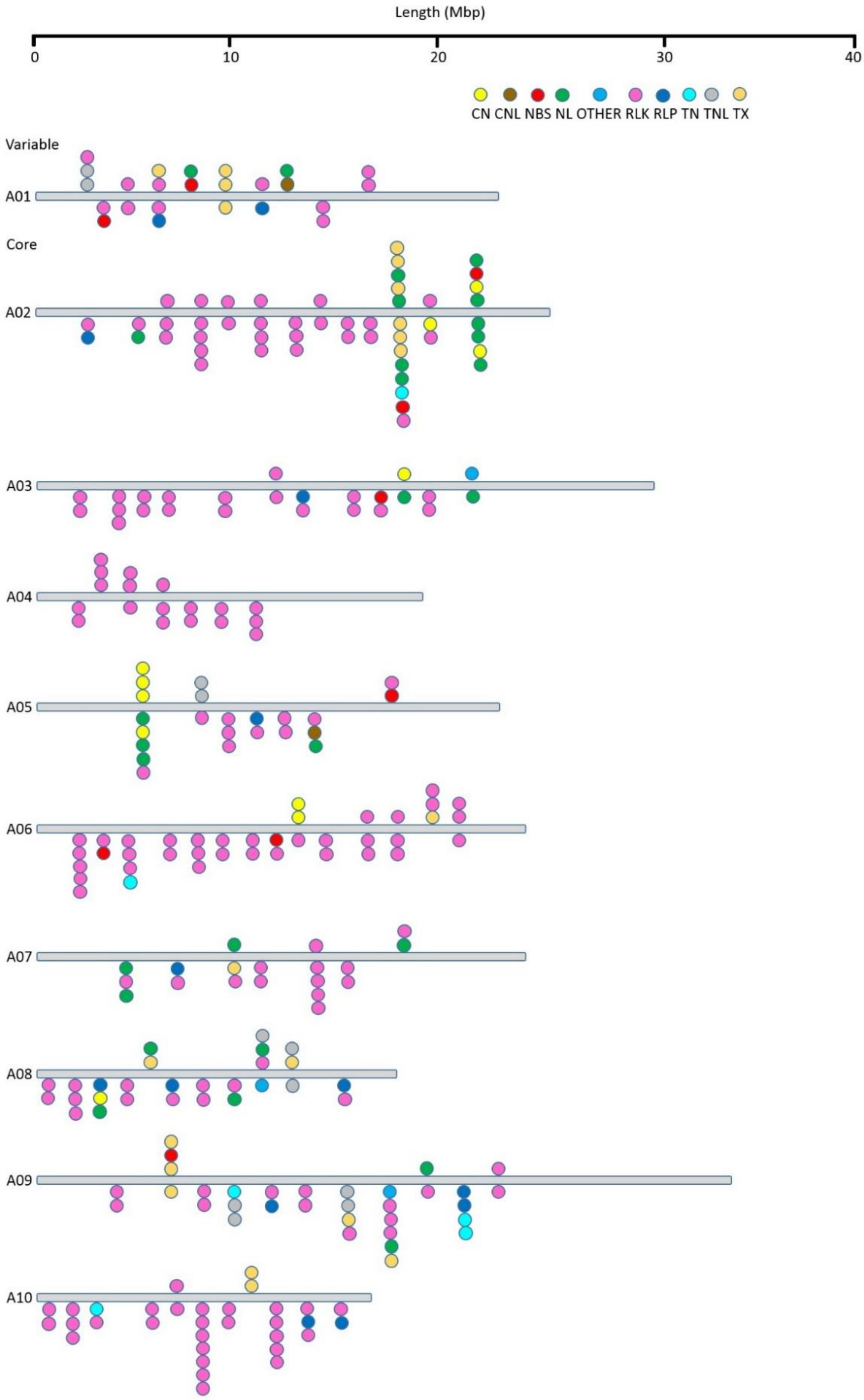

Supplementary Figure 5: Physical clustering of TM-LRR genes on the chromosome of the A genome of *B. napus*. The colourful circles above (variable) and below (core) each chromosome (grey bars) are designated for NBS classes. Chromosome lengths are shown in megabase pairs on the scale at the top.

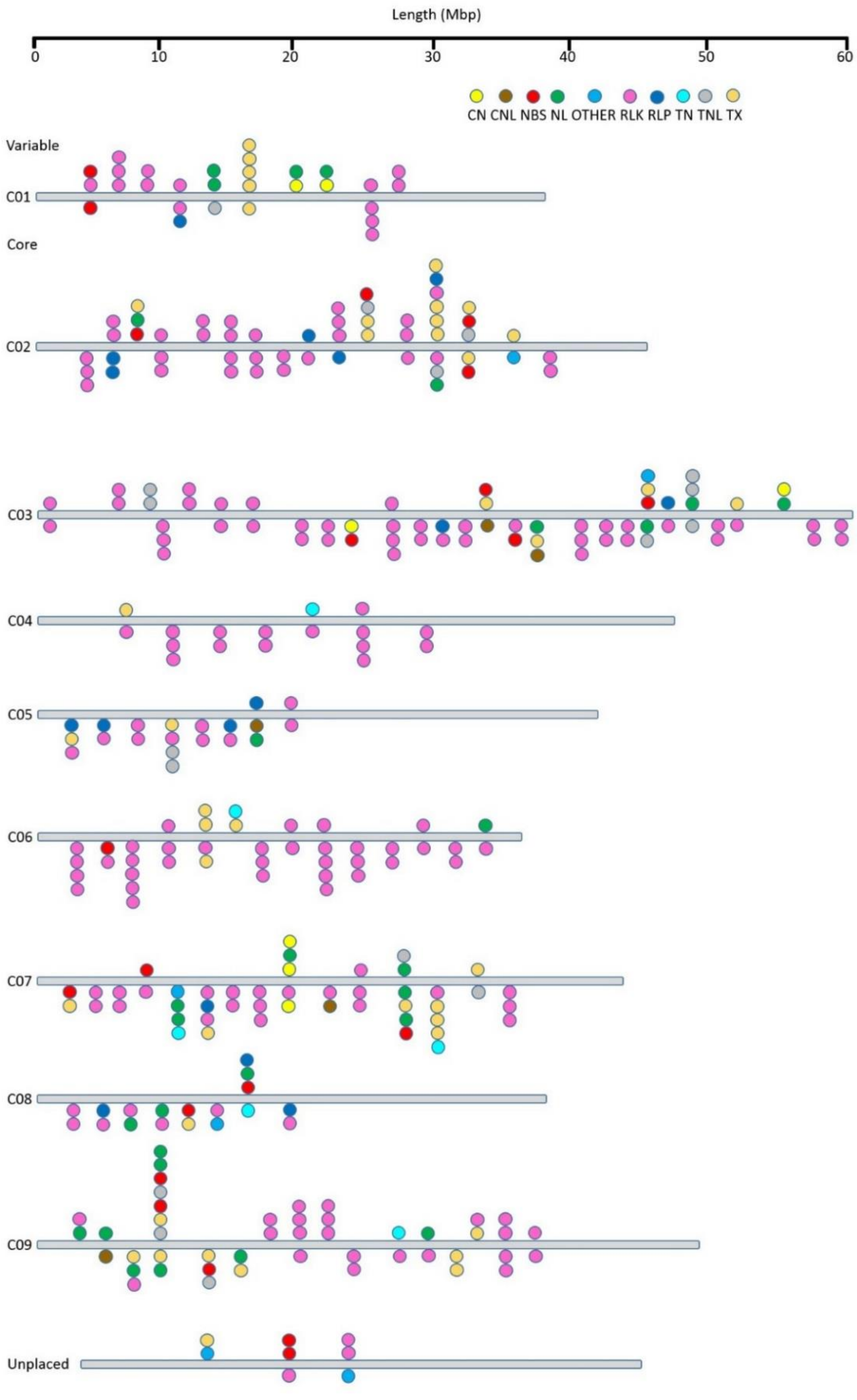

Supplementary Figure 6: Physical clustering of TM-LRR genes on the chromosome of the C genome of *B. napus*. The colourful circles above (variable) and below (core) each chromosome (grey bars) are designated for NBS classes. Chromosome lengths are shown in megabase pairs on the scale at the top.

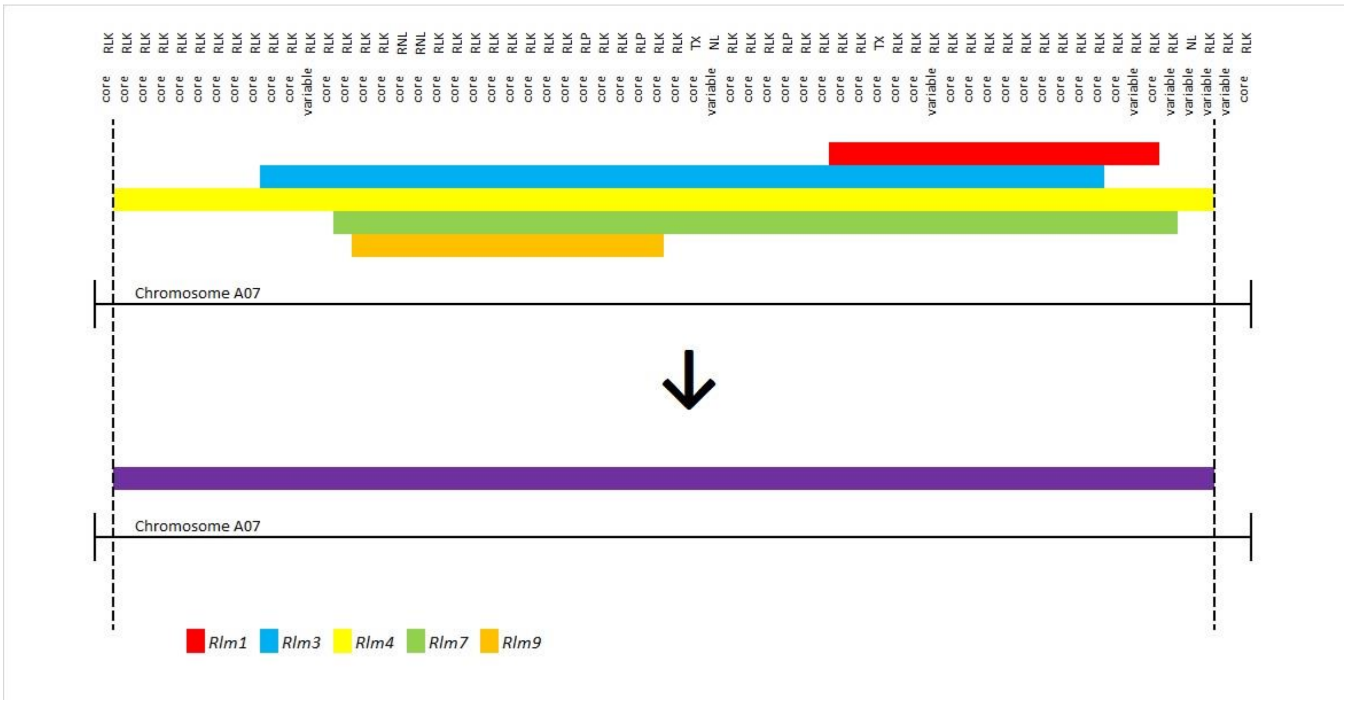

Supplementary Figure 7: *Rlm1*, *Rlm3*, *Rlm4*, *Rlm7* and *Rlm9* QTL were combined into non-redundant QTL regions by combining QTL length overlaps into single contiguous regions. Non-QTL regions contain no QTL whatsoever.
