# Supplementary material for: Characterization of disease resistance genes in the Brassica napus pangenome reveals significant structural variation
Source: Plant Biotechnol J. 2019 Oct 10;18(4):969–82. doi: 10.1111/pbi.13262 (PMC7061875; doi:10.1111/pbi.13262)
Supplement: Supplementary file 3 — Table S2 Morphotype/lines that harbour at least one RGA of each class in the pangenome. Table S3 The number of lost genes in the pangenome additional contigs, reference genome and reference genome unplaced contigs. Table S4 The number of SNPs in core and variable genes in the reference genome, pangenome additional contigs and reference genome unplaced contigs. Table S5 The numbers of non‐synonymous, synonymous and total SNPs on core and variable R‐genes. Table S6 The numbers of non‐synonymous and synonymous SNPs, mis‐sense and non‐sense variants and other effects in different RGAs. Table S7 RGA candidates underlying reported QTL for blackleg in the Darmor v 8.1 assembly. [file PBI-18-969-s001.pdf]

Supplementary Table 2: Morphotype/lines that harbour at least one RGA of each.

Morphotypes	Lines	RLK	RLP	NL	TX	NBS	TNL	CNL	CN	TN	OTHER	RNL	RN
		(■)	(▲)	(●)	(■)	(▲)	(●)	(■)	(▲)	(●)	(■)	(▲)	(●)
Non-synthetic (Fodder)	English_Giant_194	■	▲	●	■	▲	●	■	▲	●			●
	Nunsdale	■	▲	●	■	▲	●	■	▲	●	■		●
	Palu	■	▲	●	■	▲	●	■	▲	●			●
Non- Synthetic (Swede)	Fortin	■	▲	●		▲	●		▲	●			
	Sensation	■	▲	●	■	▲	●	■	▲	●			●
Non-synthetic (Vegetable)	Chuosenshu	■	▲	●	■	▲	●	■	▲	●			
	GSchnittkohl	■	▲	●	■	▲	●	■	▲	●			●
Non-synthetic (Oilseed)	ABUKUMA_NATANE	■	▲	●	■	▲	●	■	▲	●			
	Alaska	■	▲	●		▲	●	■	▲	●			
	Aragon	■	▲	●	■	▲	●		▲	●			●
	Beluga	■	▲	●	■	▲	●	■	▲				
	Canberra x CourageDH	■	▲	●	■	▲	●		▲	●			
	Darmor	■	▲	●		▲	●		▲				
	DH5	■	▲	●		▲	●	■	▲	●			
	Dippes	■	▲	●	■	▲	●	■	▲	●			
	E94197	■	▲	●	■	▲	●		▲	●			
	EVVIN	■	▲	●	■	▲	●	■	▲	●			●
	Expert	■	▲	●	■	▲	●		▲	●			
	GLuesewitzer	■	▲	●	■	▲	●	■	▲	●			●
	Jupiter	■	▲	●	■	▲	●	■	▲	●			
	Kromerska	■	▲	●	■	▲	●	■	▲	●			●
	Major	■	▲	●	■	▲	●		▲	●			
	MSL007C	■	▲	●	■	▲	●	■	▲	●			●
	Olimpiade	■	▲	●	■	▲	●	■	▲	●			
	Pacific	■	▲	●	■	▲	●		▲	●			
	Pirola	■	▲	●	■	▲	●		▲	●			
	Rapid	■	▲	●	■	▲	●	■	▲	●			
	Savannah	■	▲	●	■	▲	●	■	▲	●			
	Vivol	■	▲	●	■	▲	●		▲	●			
	Wotan	■	▲	●	■	▲	●	■	▲	●			
	Tapidor	■	▲	●	■	▲	●	■	▲	●			
Synthetic	CRY_1	■	▲	●	■	▲	●	■	▲	●	■		●
	G50	■	▲	●	■	▲	●	■	▲	●			●
	H149	■	▲	●	■	▲	●	■	▲	●	■		
	H165	■	▲	●	■	▲	●	■	▲	●			●
	H176	■	▲	●	■	▲	●	■	▲	●	■		●
	H44	■	▲	●	■	▲	●	■	▲	●	■		
	HIY_1	■	▲	●	■	▲	●	■	▲	●			●
	MOY_4	■	▲	●	■	▲	●	■	▲	●	■		●
	OLL1	■	▲	●	■	▲	●	■	▲	●			
	R_13_6	■	▲	●	■	▲	●	■	▲	●	■		
	R53	■	▲	●	■	▲	●	■	▲	●			●
	R76	■	▲	●	■	▲	●	■	▲	●			●
	Resyn_Go_S4	■	▲	●	■	▲	●	■	▲	●			
	RS_10_7	■	▲	●	■	▲	●	■	▲	●	■		●
	RS_4_6	■	▲	●	■	▲	●	■	▲	●	■		
	RS_7_6	■	▲	●	■	▲	●	■	▲	●	■		●
	RS_8_6	■	▲	●	■	▲	●	■	▲	●	■		
	S_13	■	▲	●	■	▲	●	■	▲	●			●
	S_39	■	▲	●	■	▲	●	■	▲	●			

Supplementary Table 3: The number of lost genes in the pangenome additional contigs, reference genome and reference genome unplaced contigs

Morphotypes	Lines	Pangenome additional contigs	Reference genome	Reference genome unplaced contigs
Non-synthetic (Fodder)	English_Giant_194	271	21	1
	Nunsdale	233	6	1
	Palu	264	18	1
	**Average**	**256**	**15**	**1**
Non- Synthetic (Swede)	Fortin	298	8	0
	Sensation	233	60	2
	**Average**	**266**	**34**	**1**
Non-synthetic (Vegetable)	Chuosenshu	273	24	2
	GSchnittkohl	260	39	2
	**Average**	**267**	**32**	**2**
Non-synthetic (Oilseed)	ABUKUMA_NATANE	267	35	1
	Alaska	299	15	0
	Aragon	281	31	0
	Beluga	295	12	0
	Canberra x CourageDH	297	23	0
	Darmor	344	0	0
	DH5	300	14	0
	Dippes	265	26	0
	E94197	288	16	0
	EVVIN	250	28	3
	Expert	302	11	2
	GLuesewitzer	246	25	0
	Jupiter	274	22	0
	Kromerska	242	29	0
	Major	279	23	0
	MSL007C	273	11	1
	Olimpiade	258	15	1
	Pacific	290	19	0
	Pirola	290	25	2
	Rapid	257	21	0
	Savannah	275	30	0
	Vivol	291	18	1
	Wotan	289	29	0
	Tapidor	276	27	1
	**Total**	**8,560**	**681**	**21**
	**Average**	**281**	**22**	**1**
Synthetic	CRY_1	159	53	2
	G50	261	33	5
	H149	258	50	3
	H165	239	74	0
	H176	263	19	1
	H44	179	63	2
	HIY_1	180	35	4
	MOY_4	164	53	4
	OLL1	172	6	0
	R_13_6	160	30	0
	R53	234	74	4
	R76	196	73	3
	Resyn_Go_S4	267	20	0
	RS_10_7	180	71	1
	RS_4_6	178	67	3
	RS_7_6	164	34	0
	RS_8_6	220	36	0
	S_13	281	28	1
	S_39	255	56	0
	**Total**	**4,010**	**875**	**33**
	**Average**	**212**	**47**	**2**
	**Total**	**12,570**	**1,556**	**54**

Supplementary Table 4: The number of SNPs in core and variable genes in the reference genome, pangenome additional contigs and reference genome unplaced contigs

	Presence/absence status	Number of R-gene with SNP	Number of SNP
R-genes with SNP	Core	731	10,584
	Variable	299	4,734
	**Total**	**1,030**	**15,318**
R-genes without SNP	Core	265	0
	Variable	454	0
	**Total**	**719**	0
Total number of R-genes	Core	996	10,584
	Variable	753	4734
	**Total**	**1,749**	**15,318**

	Pangenome contigs	Number of R-gene with SNP (core-variable)	Number of SNP
Reference genome	A genome	505 (396-109)	7,793
	C genome	466 (324-142)	6,674
	**A and C**	**971 (720-251)**	**14,467**
Pangenome additional contigs		37 (0-37)	594
Reference genome unplaced contigs		22 (11-11)	257
	**Total**	**1,030 (731-299)**	**15,318**

Supplementary Table 5: The numbers of non-synonymous, synonymous and total SNPs on core and variable R-genes

Gene	Class	PAV	non-synonymous	synonymous	Total SNP
BnaA01g03300.1D2	RLK	core	11	8	19
BnaA01g03380.1D2	RN	core	9	4	13
BnaA01g04330.1D2	RLK	core	7	3	10
BnaA01g05390.1D2	RLK	core	6	0	6
BnaA01g05910.1D2	RLK	core	12	7	19
BnaA01g06120.1D2	RLK	core	1	0	1
BnaA01g06880.1D2	RLK	core	23	5	28
BnaA01g08130.1D2	RLP	core	4	2	6
BnaA01g08780.1D2	RLK	core	13	3	16
BnaA01g09110.1D2	RLK	core	1	0	1
BnaA01g12510.1D2	TX	core	10	3	13
BnaA01g12750.1D2	RLK	core	13	11	24
BnaA01g13880.1D2	RLK	core	21	14	35
BnaA01g17020.1D2	RLK	core	7	2	9
BnaA01g20350.1D2	RLP	core	1	0	1
BnaA01g22120.1D2	RLK	core	4	2	6
BnaA01g23510.1D2	RLK	core	4	4	8
BnaA01g26380.1D2	RLK	core	5	3	8
BnaA01g27450.1D2	RLK	core	6	2	8
BnaA01g27710.1D2	RLK	core	4	0	4
BnaA01g34440.1D2	RLK	core	7	5	12
BnaA01g34850.1D2	RLK	core	84	47	131
BnaA02g01110.1D2	RLK	core	2	0	2
BnaA02g03060.1D2	RLK	core	16	14	30
BnaA02g04210.1D2	RLK	core	10	9	19
BnaA02g04280.1D2	NL	core	18	8	26
BnaA02g05300.1D2	RLK	core	9	6	15
BnaA02g05780.1D2	RLK	core	16	12	28
BnaA02g06200.1D2	RLK	core	4	2	6
BnaA02g07400.1D2	RLK	core	21	11	32
BnaA02g07410.1D2	RLK	core	44	28	72
BnaA02g07880.1D2	RLK	core	31	18	49
BnaA02g07890.1D2	RLK	core	1	0	1
BnaA02g07930.1D2	RLK	core	7	3	10
BnaA02g07940.1D2	RLK	core	1	0	1
BnaA02g09640.1D2	RLK	core	14	9	23
BnaA02g13290.1D2	RLP	core	6	5	11
BnaA02g13760.1D2	RLK	core	5	3	8
BnaA02g13790.1D2	RLK	core	8	6	14
BnaA02g13870.1D2	RLK	core	1	1	2
BnaA02g15810.1D2	RLK	core	10	2	12
BnaA02g15820.1D2	RLK	core	10	4	14
BnaA02g15890.1D2	RLK	core	6	4	10
BnaA02g16960.1D2	NL	core	3	1	4
BnaA02g20380.1D2	RLK	core	1	1	2
BnaA02g20610.1D2	RLK	core	9	6	15
BnaA02g21890.1D2	RLK	core	4	2	6
BnaA02g22210.1D2	RLK	core	4	2	6
BnaA02g22280.1D2	RLK	core	21	8	29
BnaA02g24180.1D2	TX	core	6	4	10
BnaA02g24200.1D2	TX	core	10	8	18

BnaA02g24240.1D2	NBS	core	4	2	6
BnaA02g24300.1D2	TN	core	11	5	16
BnaA02g24410.1D2	CN	core	1	0	1
BnaA02g24510.1D2	RLK	core	5	4	9
BnaA02g24730.1D2	RLP	core	1	1	2
BnaA02g26190.1D2	RLK	core	6	1	7
BnaA02g30520.1D2	RLK	core	11	8	19
BnaA02g30810.1D2	NL	core	13	4	17
BnaA02g31870.1D2	NL	core	27	7	34
BnaA02g31900.1D2	CN	core	16	3	19
BnaA02g31970.1D2	NL	core	20	4	24
BnaA03g00040.1D2	RLK	core	9	5	14
BnaA03g01380.1D2	RLK	core	13	13	26
BnaA03g02700.1D2	RLK	core	2	0	2
BnaA03g04350.1D2	RLK	core	5	5	10
BnaA03g05240.1D2	RLK	core	56	35	91
BnaA03g06060.1D2	RLK	core	6	3	9
BnaA03g07060.1D2	RLK	core	11	7	18
BnaA03g07410.1D2	RLK	core	18	17	35
BnaA03g07980.1D2	RLK	core	2	0	2
BnaA03g09870.1D2	RLK	core	3	1	4
BnaA03g09880.1D2	RLK	core	32	25	57
BnaA03g09890.1D2	RLK	core	15	14	29
BnaA03g10560.1D2	RLK	core	12	5	17
BnaA03g12460.1D2	RLK	core	21	19	40
BnaA03g12540.1D2	RLK	core	2	0	2
BnaA03g12930.1D2	RLK	core	8	4	12
BnaA03g14450.1D2	RLK	core	5	2	7
BnaA03g15720.1D2	RLK	core	5	2	7
BnaA03g16540.1D2	RLK	core	6	4	10
BnaA03g17020.1D2	RLK	core	5	4	9
BnaA03g17220.1D2	RLK	core	38	31	69
BnaA03g17560.1D2	RLK	core	5	4	9
BnaA03g18630.1D2	RLK	core	22	14	36
BnaA03g20890.1D2	RLK	core	3	0	3
BnaA03g22960.1D2	RLK	core	19	9	28
BnaA03g24240.1D2	RLK	core	12	9	21
BnaA03g26050.1D2	RLK	core	3	1	4
BnaA03g26250.1D2	RLK	core	1	0	1
BnaA03g27970.1D2	RLK	core	13	6	19
BnaA03g28010.1D2	RLP	core	3	0	3
BnaA03g28760.1D2	RLP	core	43	6	49
BnaA03g34200.1D2	RLK	core	10	10	20
BnaA03g35020.1D2	RLK	core	1	1	2
BnaA03g35900.1D2	RLP	core	18	6	24
BnaA03g36180.1D2	RLP	core	4	1	5
BnaA03g37010.1D2	RLK	core	1	0	1
BnaA03g37740.1D2	RLK	core	55	30	85
BnaA03g38520.1D2	RLK	core	1	0	1
BnaA03g39080.1D2	RLK	core	4	0	4
BnaA03g40010.1D2	RLK	core	8	4	12
BnaA03g42720.1D2	NL	core	46	31	77
BnaA03g43450.1D2	RLK	core	10	2	12

BnaA03g44490.1D2	TN	core	8	4	12
BnaA03g45870.1D2	RLK	core	2	2	4
BnaA03g46970.1D2	NL	core	6	2	8
BnaA03g48090.1D2	RLK	core	6	1	7
BnaA03g48820.1D2	RLK	core	17	12	29
BnaA03g48960.1D2	RLK	core	1	0	1
BnaA03g49800.1D2	RLK	core	13	12	25
BnaA03g50530.1D2	RLK	core	11	6	17
BnaA03g52260.1D2	RLK	core	7	2	9
BnaA04g02560.1D2	RLK	core	3	2	5
BnaA04g03210.1D2	RLK	core	9	6	15
BnaA04g03400.1D2	RLK	core	3	2	5
BnaA04g04610.1D2	RLK	core	15	12	27
BnaA04g08140.1D2	RLK	core	9	7	16
BnaA04g08320.1D2	RLK	core	1	1	2
BnaA04g08330.1D2	RLK	core	16	8	24
BnaA04g09910.1D2	RLK	core	1	0	1
BnaA04g10380.1D2	RLK	core	25	13	38
BnaA04g12740.1D2	RLK	core	18	10	28
BnaA04g13010.1D2	RLK	core	19	13	32
BnaA04g13130.1D2	RLK	core	7	3	10
BnaA04g16170.1D2	RLK	core	1	0	1
BnaA04g16180.1D2	RLK	core	20	8	28
BnaA04g17010.1D2	RLK	core	16	11	27
BnaA04g17920.1D2	RLK	core	1	0	1
BnaA04g18770.1D2	RLK	core	11	5	16
BnaA04g19050.1D2	RLK	core	7	4	11
BnaA04g23300.1D2	RLK	core	22	18	40
BnaA04g23350.1D2	RLK	core	4	4	8
BnaA04g23410.1D2	RLK	core	10	8	18
BnaA04g23630.1D2	RLK	core	2	2	4
BnaA04g23770.1D2	RLK	core	34	19	53
BnaA04g24750.1D2	RLK	core	2	2	4
BnaA05g02830.1D2	RLP	core	16	11	27
BnaA05g04800.1D2	RLK	core	6	1	7
BnaA05g05430.1D2	RLP	core	40	12	52
BnaA05g06820.1D2	RLK	core	2	0	2
BnaA05g07310.1D2	RLK	core	16	13	29
BnaA05g07820.1D2	CN	core	4	3	7
BnaA05g07860.1D2	NL	core	4	2	6
BnaA05g07960.1D2	RLK	core	35	23	58
BnaA05g08790.1D2	RLK	core	8	7	15
BnaA05g09970.1D2	RLK	core	2	2	4
BnaA05g12430.1D2	RLK	core	9	6	15
BnaA05g12830.1D2	RLK	core	1	0	1
BnaA05g13150.1D2	RLK	core	7	4	11
BnaA05g13380.1D2	RLK	core	10	3	13
BnaA05g15590.1D2	RLK	core	4	2	6
BnaA05g15940.1D2	RLK	core	7	0	7
BnaA05g15960.1D2	RLK	core	28	10	38
BnaA05g15970.1D2	RLK	core	33	7	40
BnaA05g16310.1D2	RLP	core	2	0	2
BnaA05g16340.1D2	RLK	core	2	1	3

BnaA05g20470.1D2	RLK	core	6	3	9
BnaA05g22010.1D2	RLK	core	12	6	18
BnaA05g22440.1D2	RLK	core	7	4	11
BnaA05g24480.1D2	RLK	core	17	7	24
BnaA05g25750.1D2	CNL	core	1	0	1
BnaA05g25760.1D2	NL	core	1	0	1
BnaA05g25860.1D2	RLK	core	23	14	37
BnaA05g26640.1D2	RLK	core	24	18	42
BnaA05g26920.1D2	RLK	core	6	4	10
BnaA05g29460.1D2	RLK	core	10	7	17
BnaA05g30180.1D2	RLK	core	18	12	30
BnaA06g00610.1D2	RLK	core	4	2	6
BnaA06g02020.1D2	RLK	core	12	4	16
BnaA06g02040.1D2	RLK	core	1	0	1
BnaA06g02050.1D2	RLK	core	1	1	2
BnaA06g02070.1D2	RLK	core	4	2	6
BnaA06g02140.1D2	RLK	core	19	9	28
BnaA06g03000.1D2	RLK	core	14	6	20
BnaA06g06140.1D2	RLP	core	4	4	8
BnaA06g06260.1D2	RLK	core	4	3	7
BnaA06g06430.1D2	RLK	core	2	1	3
BnaA06g06770.1D2	RLK	core	45	33	78
BnaA06g08040.1D2	RLK	core	7	5	12
BnaA06g09180.1D2	RLK	core	4	4	8
BnaA06g11450.1D2	RLK	core	16	12	28
BnaA06g11670.1D2	RLK	core	2	1	3
BnaA06g11700.1D2	TN	core	1	0	1
BnaA06g11800.1D2	RLK	core	2	1	3
BnaA06g11810.1D2	RLK	core	4	1	5
BnaA06g12130.1D2	RLK	core	11	7	18
BnaA06g13220.1D2	RLK	core	12	1	13
BnaA06g15170.1D2	RLP	core	21	12	33
BnaA06g17220.1D2	RLK	core	4	1	5
BnaA06g17230.1D2	RLK	core	20	10	30
BnaA06g17630.1D2	RLK	core	2	1	3
BnaA06g17980.1D2	RLK	core	2	1	3
BnaA06g17990.1D2	RLK	core	2	0	2
BnaA06g18000.1D2	RLK	core	3	2	5
BnaA06g20160.1D2	RLK	core	17	7	24
BnaA06g20220.1D2	RLK	core	2	2	4
BnaA06g20750.1D2	RLK	core	13	7	20
BnaA06g22390.1D2	RLK	core	16	9	25
BnaA06g22790.1D2	RLK	core	15	12	27
BnaA06g23450.1D2	RLK	core	17	10	27
BnaA06g24930.1D2	RLK	core	13	7	20
BnaA06g26120.1D2	RLK	core	15	10	25
BnaA06g26570.1D2	RLK	core	5	4	9
BnaA06g26850.1D2	RLK	core	4	3	7
BnaA06g27160.1D2	RLK	core	11	5	16
BnaA06g28360.1D2	RN	core	21	13	34
BnaA06g28440.1D2	RLK	core	13	7	20
BnaA06g28800.1D2	RLK	core	13	9	22
BnaA06g30330.1D2	RLK	core	4	4	8

BnaA06g30920.1D2	RLK	core	21	13	34
BnaA06g31200.1D2	RLK	core	16	9	25
BnaA06g32400.1D2	RLK	core	11	7	18
BnaA06g32570.1D2	RLK	core	5	3	8
BnaA06g32820.1D2	NL	core	27	7	34
BnaA06g32960.1D2	OTHER	core	3	1	4
BnaA06g33320.1D2	RLK	core	1	1	2
BnaA06g33600.1D2	RLK	core	11	10	21
BnaA06g34700.1D2	RLK	core	10	9	19
BnaA06g35180.1D2	RLK	core	8	6	14
BnaA06g35550.1D2	RLK	core	20	16	36
BnaA06g36740.1D2	RLK	core	1	1	2
BnaA06g36820.1D2	RLK	core	3	2	5
BnaA06g38010.1D2	RLK	core	13	5	18
BnaA06g38150.1D2	RLK	core	15	11	26
BnaA06g38810.1D2	RLK	core	10	8	18
BnaA07g00170.1D2	RLK	core	4	1	5
BnaA07g01730.1D2	RLK	core	1	0	1
BnaA07g02390.1D2	RLK	core	2	2	4
BnaA07g03220.1D2	RLK	core	7	3	10
BnaA07g03430.1D2	RLK	core	1	0	1
BnaA07g05410.1D2	RLK	core	10	9	19
BnaA07g07090.1D2	RLK	core	13	7	20
BnaA07g07350.1D2	RLK	core	8	5	13
BnaA07g08230.1D2	RLK	core	1	1	2
BnaA07g11390.1D2	RLK	core	4	0	4
BnaA07g11510.1D2	RLK	core	8	5	13
BnaA07g11690.1D2	RNL	core	27	2	29
BnaA07g11700.1D2	RNL	core	25	10	35
BnaA07g11760.1D2	RLK	core	3	2	5
BnaA07g12580.1D2	RLK	core	12	8	20
BnaA07g12710.1D2	RLK	core	17	10	27
BnaA07g13780.1D2	RLK	core	11	3	14
BnaA07g13890.1D2	RLK	core	2	1	3
BnaA07g14090.1D2	RLK	core	17	7	24
BnaA07g14670.1D2	RLK	core	14	10	24
BnaA07g16010.1D2	RLK	core	21	15	36
BnaA07g16020.1D2	RLP	core	4	4	8
BnaA07g16760.1D2	RLK	core	6	3	9
BnaA07g16990.1D2	RLK	core	23	14	37
BnaA07g17170.1D2	RLP	core	10	5	15
BnaA07g17390.1D2	RLK	core	1	1	2
BnaA07g21540.1D2	TX	core	1	0	1
BnaA07g21660.1D2	RLK	core	3	2	5
BnaA07g21740.1D2	RLK	core	12	9	21
BnaA07g21960.1D2	RLK	core	25	21	46
BnaA07g22730.1D2	RLP	core	5	2	7
BnaA07g23630.1D2	RLK	core	6	2	8
BnaA07g24060.1D2	RLK	core	27	21	48
BnaA07g25020.1D2	RLK	core	8	8	16
BnaA07g25230.1D2	TX	core	4	1	5
BnaA07g25570.1D2	RLK	core	3	1	4
BnaA07g25630.1D2	RLK	core	13	3	16

BnaA07g25640.1D2	RLK	core	14	9	23
BnaA07g25910.1D2	RLK	core	1	1	2
BnaA07g26080.1D2	RLK	core	9	7	16
BnaA07g27920.1D2	RLK	core	2	0	2
BnaA07g28050.1D2	RLK	core	2	1	3
BnaA07g29090.1D2	RLK	core	8	8	16
BnaA07g29360.1D2	RLK	core	3	2	5
BnaA07g29470.1D2	RLK	core	8	4	12
BnaA07g35400.1D2	RLK	core	3	1	4
BnaA08g00080.1D2	RLK	core	6	3	9
BnaA08g00090.1D2	RLK	core	3	1	4
BnaA08g00880.1D2	RLK	core	25	17	42
BnaA08g01300.1D2	RLK	core	15	12	27
BnaA08g01400.1D2	RLK	core	7	4	11
BnaA08g01870.1D2	RLK	core	11	8	19
BnaA08g02170.1D2	NL	core	1	0	1
BnaA08g02730.1D2	RLK	core	3	2	5
BnaA08g03110.1D2	RLK	core	2	2	4
BnaA08g04910.1D2	RLK	core	15	9	24
BnaA08g06080.1D2	RLK	core	1	1	2
BnaA08g08830.1D2	RLK	core	5	5	10
BnaA08g09020.1D2	RLK	core	10	3	13
BnaA08g10430.1D2	RLK	core	4	3	7
BnaA08g11280.1D2	RLK	core	4	3	7
BnaA08g11350.1D2	RNL	core	16	13	29
BnaA08g11730.1D2	RLK	core	6	1	7
BnaA08g12220.1D2	RLK	core	15	13	28
BnaA08g13070.1D2	RLK	core	5	5	10
BnaA08g13260.1D2	RLK	core	1	1	2
BnaA08g14930.1D2	OTHER	core	7	5	12
BnaA08g16230.1D2	RLK	core	2	1	3
BnaA08g17840.1D2	RLP	core	7	4	11
BnaA08g19610.1D2	TNL	core	23	9	32
BnaA08g20250.1D2	RLP	core	3	0	3
BnaA08g20440.1D2	RLP	core	7	4	11
BnaA08g22160.1D2	NBS	core	4	3	7
BnaA08g24440.1D2	RLK	core	1	1	2
BnaA08g24730.1D2	RLK	core	2	2	4
BnaA08g25310.1D2	RLK	core	1	0	1
BnaA08g26030.1D2	RLK	core	15	11	26
BnaA08g27540.1D2	RLK	core	10	5	15
BnaA09g05700.1D2	RLK	core	6	4	10
BnaA09g05910.1D2	RLK	core	5	4	9
BnaA09g06170.1D2	RLK	core	20	9	29
BnaA09g07060.1D2	RLK	core	7	6	13
BnaA09g10420.1D2	RLK	core	10	7	17
BnaA09g10740.1D2	RLK	core	1	1	2
BnaA09g11380.1D2	RLK	core	12	10	22
BnaA09g12030.1D2	RLK	core	11	8	19
BnaA09g13320.1D2	RLK	core	10	4	14
BnaA09g13690.1D2	TX	core	2	2	4
BnaA09g15280.1D2	RLK	core	2	2	4
BnaA09g15680.1D2	RLK	core	1	1	2

BnaA09g16330.1D2	RLP	core	2	1	3
BnaA09g17610.1D2	RLK	core	14	9	23
BnaA09g19780.1D2	RLK	core	47	23	70
BnaA09g20690.1D2	TNL	core	3	0	3
BnaA09g21360.1D2	RLK	core	1	1	2
BnaA09g24240.1D2	TNL	core	12	6	18
BnaA09g26110.1D2	RLK	core	6	4	10
BnaA09g26980.1D2	RLK	core	2	2	4
BnaA09g27070.1D2	RLP	core	2	2	4
BnaA09g28250.1D2	RLK	core	12	5	17
BnaA09g29330.1D2	TNL	core	5	3	8
BnaA09g29340.1D2	TNL	core	35	14	49
BnaA09g29350.1D2	RLK	core	11	10	21
BnaA09g31150.1D2	RLK	core	3	3	6
BnaA09g31750.1D2	CNL	core	1	1	2
BnaA09g32190.1D2	NL	core	17	9	26
BnaA09g32200.1D2	TX	core	4	0	4
BnaA09g32210.1D2	OTHER	core	3	2	5
BnaA09g32300.1D2	RLK	core	20	10	30
BnaA09g32330.1D2	RLK	core	23	14	37
BnaA09g33570.1D2	RLK	core	2	1	3
BnaA09g34730.1D2	RLK	core	15	3	18
BnaA09g35580.1D2	RLK	core	1	0	1
BnaA09g35730.1D2	RLK	core	7	3	10
BnaA09g35940.1D2	RLK	core	7	5	12
BnaA09g36400.1D2	RLK	core	10	7	17
BnaA09g37280.1D2	RLK	core	27	22	49
BnaA09g37950.1D2	RLK	core	14	14	28
BnaA09g38530.1D2	RLK	core	5	1	6
BnaA09g40970.1D2	RLK	core	9	8	17
BnaA09g42770.1D2	RLK	core	3	1	4
BnaA09g44270.1D2	RLK	core	3	2	5
BnaA09g45370.1D2	TN	core	2	0	2
BnaA09g45380.1D2	RLP	core	15	7	22
BnaA09g46110.1D2	RLK	core	4	3	7
BnaA09g48680.1D2	RLK	core	1	1	2
BnaA09g52120.1D2	RLK	core	15	13	28
BnaA10g06390.1D2	RLK	core	4	1	5
BnaA10g06440.1D2	RLK	core	8	3	11
BnaA10g07140.1D2	RLK	core	1	1	2
BnaA10g07390.1D2	RLK	core	1	0	1
BnaA10g07400.1D2	RLK	core	8	2	10
BnaA10g07410.1D2	RLK	core	1	0	1
BnaA10g07650.1D2	RLK	core	4	3	7
BnaA10g09120.1D2	RLK	core	1	1	2
BnaA10g09460.1D2	TN	core	1	0	1
BnaA10g09500.1D2	RLK	core	1	1	2
BnaA10g09870.1D2	RLK	core	17	13	30
BnaA10g10000.1D2	TN	core	3	3	6
BnaA10g10380.1D2	RLK	core	10	4	14
BnaA10g10430.1D2	RLK	core	2	2	4
BnaA10g11930.1D2	RLK	core	17	10	27
BnaA10g12560.1D2	RLK	core	9	3	12

BnaA10g12830.1D2	RLK	core	6	4	10
BnaA10g12880.1D2	RLK	core	6	4	10
BnaA10g12900.1D2	RLK	core	5	4	9
BnaA10g12950.1D2	RLK	core	1	0	1
BnaA10g13610.1D2	OTHER	core	22	8	30
BnaA10g14170.1D2	RLK	core	8	5	13
BnaA10g14640.1D2	RLK	core	1	0	1
BnaA10g14660.1D2	RLK	core	2	1	3
BnaA10g14840.1D2	RLK	core	19	9	28
BnaA10g17590.1D2	RLP	core	7	2	9
BnaA10g17700.1D2	RLK	core	6	4	10
BnaA10g18120.1D2	RLK	core	1	1	2
BnaA10g18330.1D2	RLK	core	10	8	18
BnaA10g19190.1D2	RLK	core	28	19	47
BnaA10g19700.1D2	RLK	core	4	2	6
BnaA10g20110.1D2	RLK	core	10	10	20
BnaA10g20770.1D2	NBS	core	13	2	15
BnaA10g21730.1D2	RLK	core	9	9	18
BnaA10g23030.1D2	RLK	core	9	4	13
BnaA10g23040.1D2	RLK	core	7	4	11
BnaA10g23100.1D2	RLK	core	10	4	14
BnaA10g23120.1D2	RLK	core	29	20	49
BnaA10g23220.1D2	RLK	core	7	4	11
BnaA10g23500.1D2	RLK	core	21	15	36
BnaA10g23580.1D2	RLK	core	4	2	6
BnaA10g26650.1D2	RLK	core	4	2	6
BnaA10g26670.1D2	RLP	core	12	5	17
BnaC01g06430.1D2	RLK	core	1	1	2
BnaC01g07250.1D2	RLK	core	14	9	23
BnaC01g08730.1D2	RLK	core	10	5	15
BnaC01g08930.1D2	RLP	core	1	0	1
BnaC01g09980.1D2	RLK	core	1	0	1
BnaC01g10090.1D2	RLP	core	1	0	1
BnaC01g10550.1D2	TNL	core	28	8	36
BnaC01g10890.1D2	NL	core	1	0	1
BnaC01g12760.1D2	RLK	core	1	0	1
BnaC01g13450.1D2	RLK	core	3	2	5
BnaC01g14500.1D2	RLK	core	30	18	48
BnaC01g21160.1D2	RLK	core	1	0	1
BnaC01g22120.1D2	RLK	core	7	5	12
BnaC01g22750.1D2	RLK	core	2	1	3
BnaC01g26020.1D2	CNL	core	2	2	4
BnaC01g29880.1D2	RLK	core	1	0	1
BnaC01g33110.1D2	RLK	core	8	2	10
BnaC02g00250.1D2	RLK	core	12	8	20
BnaC02g03870.1D2	RLK	core	38	21	59
BnaC02g03940.1D2	RLK	core	19	14	33
BnaC02g03990.1D2	RLK	core	8	5	13
BnaC02g06250.1D2	RLP	core	17	5	22
BnaC02g11750.1D2	RLK	core	10	4	14
BnaC02g11760.1D2	RLK	core	3	1	4
BnaC02g15510.1D2	RLK	core	15	4	19
BnaC02g15550.1D2	RLK	core	12	8	20

BnaC02g17490.1D2	RLK	core	1	1	2
BnaC02g17510.1D2	RLK	core	1	1	2
BnaC02g17640.1D2	RLK	core	1	0	1
BnaC02g18850.1D2	RLK	core	1	0	1
BnaC02g20350.1D2	RLP	core	3	2	5
BnaC02g29700.1D2	TNL	core	61	23	84
BnaC02g29900.1D2	RLK	core	4	0	4
BnaC02g30130.1D2	NBS	core	1	1	2
BnaC02g35430.1D2	RLK	core	5	4	9
BnaC02g39460.1D2	RLK	core	16	5	21
BnaC02g40460.1D2	RLK	core	5	1	6
BnaC02g40480.1D2	RLK	core	12	3	15
BnaC03g00190.1D2	RLK	core	4	3	7
BnaC03g06710.1D2	RLK	core	4	0	4
BnaC03g08730.1D2	RLK	core	2	1	3
BnaC03g09870.1D2	RLK	core	10	4	14
BnaC03g09880.1D2	RLK	core	5	4	9
BnaC03g09900.1D2	RLK	core	7	5	12
BnaC03g11420.1D2	RLK	core	2	2	4
BnaC03g12120.1D2	RLK	core	3	1	4
BnaC03g13080.1D2	RLK	core	12	7	19
BnaC03g14990.1D2	RLK	core	1	0	1
BnaC03g15290.1D2	RLK	core	1	1	2
BnaC03g15850.1D2	RLK	core	4	2	6
BnaC03g17400.1D2	RLK	core	6	1	7
BnaC03g17780.1D2	RLK	core	6	5	11
BnaC03g17920.1D2	RLK	core	16	10	26
BnaC03g18300.1D2	RLK	core	2	2	4
BnaC03g19250.1D2	RLK	core	3	1	4
BnaC03g19510.1D2	RLK	core	22	9	31
BnaC03g21780.1D2	RLK	core	8	5	13
BnaC03g24180.1D2	RLK	core	15	8	23
BnaC03g24370.1D2	RLK	core	3	1	4
BnaC03g25820.1D2	CN	core	6	2	8
BnaC03g25830.1D2	NBS	core	14	3	17
BnaC03g26780.1D2	NL	core	8	1	9
BnaC03g26910.1D2	RLK	core	9	4	13
BnaC03g27570.1D2	RLK	core	20	3	23
BnaC03g27590.1D2	RLK	core	1	0	1
BnaC03g27790.1D2	RLK	core	11	2	13
BnaC03g29080.1D2	RLK	core	17	6	23
BnaC03g29180.1D2	RLK	core	2	1	3
BnaC03g29800.1D2	RLK	core	6	3	9
BnaC03g29850.1D2	RLP	core	2	2	4
BnaC03g31370.1D2	RLP	core	2	1	3
BnaC03g37400.1D2	RLK	core	1	1	2
BnaC03g37830.1D2	RLK	core	1	1	2
BnaC03g38910.1D2	RLP	core	3	0	3
BnaC03g39710.1D2	CNL	core	1	0	1
BnaC03g40010.1D2	RLK	core	9	3	12
BnaC03g40420.1D2	RLK	core	1	1	2
BnaC03g40910.1D2	RLK	core	4	0	4
BnaC03g41810.1D2	TX	core	3	1	4

BnaC03g42310.1D2	CNL	core	33	8	41
BnaC03g42330.1D2	TX	core	9	1	10
BnaC03g42350.1D2	NL	core	28	14	42
BnaC03g42700.1D2	RLK	core	14	6	20
BnaC03g45700.1D2	RLK	core	6	4	10
BnaC03g46080.1D2	RLK	core	5	3	8
BnaC03g46610.1D2	RLK	core	3	2	5
BnaC03g49690.1D2	RLK	core	3	2	5
BnaC03g49740.1D2	RLK	core	2	0	2
BnaC03g50100.1D2	RLK	core	7	2	9
BnaC03g50410.1D2	RLK	core	3	1	4
BnaC03g51310.1D2	RLK	core	25	10	35
BnaC03g52950.1D2	RLK	core	9	4	13
BnaC03g53230.1D2	RLK	core	2	1	3
BnaC04g02790.1D2	RLK	core	29	19	48
BnaC04g04470.1D2	RLP	core	12	7	19
BnaC04g06770.1D2	RLK	core	3	1	4
BnaC04g08070.1D2	RLK	core	6	4	10
BnaC04g08530.1D2	RLK	core	9	5	14
BnaC04g09490.1D2	CNL	core	12	8	20
BnaC04g09900.1D2	RLK	core	4	2	6
BnaC04g10390.1D2	RLK	core	31	11	42
BnaC04g12430.1D2	RLK	core	6	1	7
BnaC04g14230.1D2	RLK	core	18	8	26
BnaC04g14400.1D2	RLK	core	2	0	2
BnaC04g16270.1D2	RLK	core	8	5	13
BnaC04g16400.1D2	RLK	core	5	2	7
BnaC04g16730.1D2	RLK	core	4	3	7
BnaC04g19830.1D2	RLK	core	26	8	34
BnaC04g20090.1D2	RLK	core	17	5	22
BnaC04g22170.1D2	RLK	core	6	1	7
BnaC04g22180.1D2	RLK	core	19	7	26
BnaC04g22190.1D2	RLK	core	2	0	2
BnaC04g22400.1D2	RLP	core	1	0	1
BnaC04g23880.1D2	RLK	core	2	1	3
BnaC04g25570.1D2	RLK	core	7	4	11
BnaC04g27940.1D2	RLK	core	7	4	11
BnaC04g28660.1D2	RLK	core	5	1	6
BnaC04g29230.1D2	RLK	core	2	0	2
BnaC04g29870.1D2	RLK	core	1	1	2
BnaC04g30810.1D2	RLK	core	1	1	2
BnaC04g32040.1D2	RLK	core	4	1	5
BnaC04g32950.1D2	RLK	core	3	1	4
BnaC04g33030.1D2	RLK	core	15	7	22
BnaC04g33090.1D2	RLK	core	2	1	3
BnaC04g33270.1D2	RLK	core	1	0	1
BnaC05g03650.1D2	RLK	core	11	6	17
BnaC05g04450.1D2	RLK	core	10	6	16
BnaC05g05020.1D2	RLK	core	12	6	18
BnaC05g07460.1D2	RLP	core	10	1	11
BnaC05g07530.1D2	RLK	core	10	6	16
BnaC05g07760.1D2	RLK	core	1	1	2
BnaC05g07810.1D2	RLP	core	17	10	27

BnaC05g07980.1D2	RLK	core	17	15	32
BnaC05g08040.1D2	RLK	core	14	7	21
BnaC05g09220.1D2	RLK	core	14	10	24
BnaC05g09630.1D2	RLP	core	18	11	29
BnaC05g10390.1D2	RLK	core	5	3	8
BnaC05g13020.1D2	RLK	core	8	5	13
BnaC05g13230.1D2	RLK	core	6	4	10
BnaC05g13520.1D2	RLK	core	15	7	22
BnaC05g14180.1D2	RLK	core	3	3	6
BnaC05g16200.1D2	RLP	core	7	3	10
BnaC05g16450.1D2	RLK	core	5	3	8
BnaC05g18680.1D2	RLK	core	3	2	5
BnaC05g18690.1D2	TNL	core	42	16	58
BnaC05g18710.1D2	TNL	core	2	2	4
BnaC05g18990.1D2	RLK	core	7	3	10
BnaC05g19770.1D2	RLK	core	1	0	1
BnaC05g20730.1D2	RLP	core	11	6	17
BnaC05g21830.1D2	RLK	core	8	3	11
BnaC05g25310.1D2	RLK	core	6	0	6
BnaC05g27670.1D2	RLK	core	5	2	7
BnaC05g30190.1D2	RLK	core	17	4	21
BnaC05g30710.1D2	RLK	core	4	0	4
BnaC05g31680.1D2	RLK	core	9	2	11
BnaC05g34680.1D2	NL	core	8	3	11
BnaC05g34810.1D2	RLK	core	5	3	8
BnaC05g35770.1D2	RLK	core	2	1	3
BnaC06g02220.1D2	RLK	core	6	1	7
BnaC06g03830.1D2	RLK	core	8	2	10
BnaC06g03940.1D2	RLK	core	7	1	8
BnaC06g03960.1D2	RLK	core	4	2	6
BnaC06g03990.1D2	RLK	core	3	1	4
BnaC06g04020.1D2	RLK	core	14	5	19
BnaC06g05420.1D2	RLK	core	5	4	9
BnaC06g07240.1D2	RLK	core	2	1	3
BnaC06g07260.1D2	RLK	core	6	0	6
BnaC06g07290.1D2	RLK	core	22	7	29
BnaC06g07750.1D2	TX	core	2	1	3
BnaC06g09570.1D2	RLK	core	6	1	7
BnaC06g09870.1D2	RLK	core	2	2	4
BnaC06g10130.1D2	RLK	core	5	2	7
BnaC06g10290.1D2	RLK	core	9	4	13
BnaC06g10860.1D2	RLK	core	20	10	30
BnaC06g12800.1D2	RLK	core	8	3	11
BnaC06g13840.1D2	RLK	core	2	1	3
BnaC06g14090.1D2	RLK	core	1	0	1
BnaC06g14650.1D2	RLK	core	9	6	15
BnaC06g17310.1D2	RLK	core	13	4	17
BnaC06g17730.1D2	RLK	core	18	6	24
BnaC06g18120.1D2	RLP	core	1	0	1
BnaC06g20080.1D2	TX	core	14	4	18
BnaC06g20570.1D2	RLK	core	4	2	6
BnaC06g20800.1D2	RLK	core	9	6	15
BnaC06g20860.1D2	RLK	core	3	2	5

BnaC06g21460.1D2	RLK	core	3	2	5
BnaC06g21880.1D2	RLK	core	1	0	1
BnaC06g23460.1D2	RLK	core	9	8	17
BnaC06g23750.1D2	RLK	core	23	5	28
BnaC06g23980.1D2	RLK	core	2	0	2
BnaC06g24020.1D2	RLK	core	9	1	10
BnaC06g24030.1D2	RLK	core	7	1	8
BnaC06g24040.1D2	RLK	core	28	11	39
BnaC06g24570.1D2	RLK	core	4	2	6
BnaC06g26600.1D2	RLK	core	8	4	12
BnaC06g26680.1D2	RLK	core	8	7	15
BnaC06g26690.1D2	RLK	core	2	1	3
BnaC06g27000.1D2	RLK	core	1	0	1
BnaC06g27040.1D2	RLK	core	4	2	6
BnaC06g28050.1D2	RLK	core	7	3	10
BnaC06g28410.1D2	RLK	core	2	1	3
BnaC06g28990.1D2	TN	core	24	12	36
BnaC06g29350.1D2	RLK	core	16	6	22
BnaC06g30030.1D2	RLK	core	4	3	7
BnaC06g34480.1D2	RLK	core	2	0	2
BnaC07g01700.1D2	TX	core	3	0	3
BnaC07g01710.1D2	NBS	core	8	3	11
BnaC07g06680.1D2	RLK	core	1	1	2
BnaC07g07280.1D2	RLK	core	5	2	7
BnaC07g07610.1D2	RLK	core	1	1	2
BnaC07g08570.1D2	RLK	core	3	0	3
BnaC07g11660.1D2	NL	core	10	2	12
BnaC07g12710.1D2	RLK	core	11	5	16
BnaC07g12940.1D2	RLK	core	1	0	1
BnaC07g13040.1D2	RLK	core	2	0	2
BnaC07g13300.1D2	OTHER	core	16	8	24
BnaC07g13860.1D2	RLK	core	3	3	6
BnaC07g14060.1D2	RLK	core	14	5	19
BnaC07g15020.1D2	NL	core	23	10	33
BnaC07g15030.1D2	OTHER	core	25	6	31
BnaC07g15050.1D2	NL	core	37	16	53
BnaC07g15060.1D2	TN	core	30	7	37
BnaC07g15180.1D2	CNL	core	34	10	44
BnaC07g15400.1D2	RLP	core	1	1	2
BnaC07g15500.1D2	RLK	core	1	0	1
BnaC07g16890.1D2	RLK	core	8	5	13
BnaC07g17610.1D2	RLK	core	26	12	38
BnaC07g17680.1D2	RLK	core	4	1	5
BnaC07g17780.1D2	RLK	core	4	2	6
BnaC07g17960.1D2	RLK	core	28	22	50
BnaC07g18210.1D2	RLK	core	1	0	1
BnaC07g18500.1D2	OTHER	core	9	4	13
BnaC07g18980.1D2	TX	core	2	0	2
BnaC07g19890.1D2	RLK	core	4	4	8
BnaC07g20520.1D2	RLK	core	2	2	4
BnaC07g21000.1D2	RLK	core	7	3	10
BnaC07g22250.1D2	RLK	core	20	11	31
BnaC07g22520.1D2	RLK	core	12	6	18

BnaC07g22610.1D2	CNL	core	22	3	25
BnaC07g25100.1D2	RLK	core	1	1	2
BnaC07g25570.1D2	RLK	core	12	5	17
BnaC07g25810.1D2	RLK	core	1	0	1
BnaC07g25880.1D2	RLK	core	3	0	3
BnaC07g26120.1D2	RLK	core	2	1	3
BnaC07g26930.1D2	RLK	core	1	0	1
BnaC07g28010.1D2	RLK	core	1	0	1
BnaC07g30880.1D2	RLK	core	6	0	6
BnaC07g31040.1D2	NL	core	33	17	50
BnaC07g31090.1D2	NL	core	71	25	96
BnaC07g31100.1D2	TX	core	8	3	11
BnaC07g31110.1D2	NBS	core	25	13	38
BnaC07g32990.1D2	TN	core	5	3	8
BnaC07g33030.1D2	TX	core	15	4	19
BnaC07g33040.1D2	TX	core	22	7	29
BnaC07g33060.1D2	TX	core	15	6	21
BnaC07g33160.1D2	RLK	core	6	2	8
BnaC07g33280.1D2	RLK	core	6	3	9
BnaC07g34150.1D2	RLK	core	2	1	3
BnaC07g34780.1D2	RLK	core	20	3	23
BnaC07g35090.1D2	NL	core	14	6	20
BnaC07g36180.1D2	RLK	core	5	3	8
BnaC07g37040.1D2	RLK	core	18	15	33
BnaC07g37360.1D2	RLK	core	3	2	5
BnaC07g37950.1D2	RLK	core	10	8	18
BnaC07g39890.1D2	NL	core	31	14	45
BnaC07g39910.1D2	TNL	core	36	9	45
BnaC07g40780.1D2	RLK	core	1	1	2
BnaC07g40820.1D2	RLK	core	5	5	10
BnaC08g01070.1D2	RLK	core	4	1	5
BnaC08g02880.1D2	RLK	core	2	0	2
BnaC08g03820.1D2	RLK	core	4	1	5
BnaC08g05480.1D2	RLK	core	4	2	6
BnaC08g05900.1D2	RLK	core	4	1	5
BnaC08g07230.1D2	RLK	core	1	0	1
BnaC08g11010.1D2	RLK	core	1	0	1
BnaC08g11480.1D2	RLK	core	1	1	2
BnaC08g11980.1D2	RLK	core	1	0	1
BnaC08g12360.1D2	RLP	core	8	6	14
BnaC08g12430.1D2	RLK	core	10	2	12
BnaC08g12580.1D2	RLK	core	25	12	37
BnaC08g14170.1D2	NL	core	20	7	27
BnaC08g15380.1D2	RLK	core	5	3	8
BnaC08g15400.1D2	NL	core	3	2	5
BnaC08g17620.1D2	RLP	core	3	1	4
BnaC08g17780.1D2	TX	core	1	1	2
BnaC08g18690.1D2	RLP	core	13	6	19
BnaC08g19640.1D2	CNL	core	1	0	1
BnaC08g20100.1D2	RLK	core	2	0	2
BnaC08g21220.1D2	RLK	core	4	1	5
BnaC08g23450.1D2	RLK	core	5	2	7
BnaC08g24510.1D2	NL	core	13	4	17

BnaC08g24910.1D2	RLK	core	10	4	14
BnaC08g27540.1D2	RLK	core	2	0	2
BnaC08g28940.1D2	RLK	core	5	5	10
BnaC08g29190.1D2	RLK	core	6	2	8
BnaC08g29950.1D2	RLK	core	5	4	9
BnaC08g33180.1D2	TN	core	1	1	2
BnaC08g33930.1D2	RLK	core	5	1	6
BnaC08g34170.1D2	RLK	core	2	2	4
BnaC08g37700.1D2	RLK	core	6	2	8
BnaC08g37800.1D2	RLP	core	15	9	24
BnaC09g00710.1D2	RLK	core	19	10	29
BnaC09g04360.1D2	RLK	core	3	1	4
BnaC09g04550.1D2	RLK	core	8	3	11
BnaC09g04960.1D2	RLK	core	28	23	51
BnaC09g05940.1D2	RLK	core	11	4	15
BnaC09g07480.1D2	OTHER	core	1	0	1
BnaC09g08500.1D2	RLK	core	16	6	22
BnaC09g09510.1D2	RLK	core	4	0	4
BnaC09g09960.1D2	RLK	core	8	6	14
BnaC09g11870.1D2	RLK	core	24	6	30
BnaC09g12810.1D2	RLK	core	3	0	3
BnaC09g15960.1D2	RLK	core	12	2	14
BnaC09g16540.1D2	NL	core	44	10	54
BnaC09g16660.1D2	RLK	core	3	1	4
BnaC09g17150.1D2	NL	core	9	4	13
BnaC09g17420.1D2	RLK	core	1	0	1
BnaC09g17580.1D2	RLK	core	7	4	11
BnaC09g18750.1D2	NBS	core	7	2	9
BnaC09g18760.1D2	TX	core	4	4	8
BnaC09g18770.1D2	TNL	core	65	18	83
BnaC09g21340.1D2	NL	core	3	3	6
BnaC09g21890.1D2	NBS	core	18	5	23
BnaC09g22160.1D2	RLK	core	4	2	6
BnaU01g00650.1D2	CN	core	3	1	4
BnaU01g03730.1D2	NL	core	8	3	11
BnaU01g07680.1D2	RLK	core	33	22	55
BnaU01g12500.1D2	NL	core	16	5	21
BnaU01g17080.1D2	RLK	core	14	7	21
BnaU01g17490.1D2	RLK	core	2	1	3
BnaU01g18170.1D2	RLK	core	11	2	13
BnaU01g22230.1D2	RLK	core	2	0	2
BnaU01g23440.1D2	OTHER	core	28	8	36
BnaU01g23970.1D2	RLK	core	13	5	18
BnaU01g27030.1D2	RLK	core	2	2	4
		**Total**	**7,027**	**3,557**	**10,584**
BnaA01g00600.1D2	RLK	variable	27	16	43
BnaA01g01380.1D2	RLK	variable	9	7	16
BnaA01g01400.1D2	TNL	variable	8	2	10
BnaA01g01410.1D2	TNL	variable	44	15	59
BnaA01g04390.1D2	RLK	variable	4	1	5
BnaA01g06470.1D2	RLK	variable	3	1	4
BnaA01g09140.1D2	RLK	variable	2	2	4
BnaA01g09250.1D2	TX	variable	2	0	2

BnaA01g09430.1D2	NL	variable	25	13	38
BnaA01g09440.1D2	NBS	variable	8	4	12
BnaA01g09640.1D2	TX	variable	13	5	18
BnaA01g11450.1D2	RLK	variable	21	9	30
BnaA01g12260.1D2	RLK	variable	14	7	21
BnaA01g15480.1D2	CN	variable	6	1	7
BnaA01g19610.1D2	CNL	variable	11	5	16
BnaA01g21730.1D2	CN	variable	2	1	3
BnaA01g23040.1D2	RLK	variable	9	6	15
BnaA01g25650.1D2	RLK	variable	4	3	7
BnaA01g25900.1D2	RLK	variable	12	7	19
BnaA01g29500.1D2	RLK	variable	3	1	4
BnaA01g30480.1D2	RLK	variable	13	6	19
BnaA01g30750.1D2	RLK	variable	6	3	9
BnaA01g31340.1D2	RLK	variable	14	7	21
BnaA01g34280.1D2	NL	variable	32	18	50
BnaA02g00540.1D2	RLK	variable	33	20	53
BnaA02g07500.1D2	RLK	variable	7	5	12
BnaA02g07870.1D2	RLK	variable	2	0	2
BnaA02g10980.1D2	RLK	variable	24	16	40
BnaA02g13830.1D2	RLK	variable	1	0	1
BnaA02g14770.1D2	RLK	variable	8	5	13
BnaA02g15610.1D2	RLK	variable	1	1	2
BnaA02g16770.1D2	RLK	variable	2	1	3
BnaA02g24250.1D2	NL	variable	4	2	6
BnaA02g24260.1D2	TX	variable	2	1	3
BnaA02g24290.1D2	NL	variable	6	2	8
BnaA02g24500.1D2	RLK	variable	1	0	1
BnaA02g26650.1D2	TX	variable	4	1	5
BnaA02g27630.1D2	TX	variable	6	2	8
BnaA02g27890.1D2	RLK	variable	4	2	6
BnaA02g28890.1D2	RLK	variable	1	1	2
BnaA02g31890.1D2	NL	variable	4	1	5
BnaA02g31920.1D2	CN	variable	11	7	18
BnaA03g00280.1D2	RLK	variable	6	3	9
BnaA03g03320.1D2	RLK	variable	42	29	71
BnaA03g06390.1D2	RLP	variable	5	1	6
BnaA03g23150.1D2	RLK	variable	5	2	7
BnaA03g25630.1D2	RLK	variable	8	5	13
BnaA03g26030.1D2	RLK	variable	4	2	6
BnaA03g42730.1D2	OTHER	variable	53	24	77
BnaA04g11840.1D2	RLK	variable	4	4	8
BnaA04g11850.1D2	RLK	variable	11	4	15
BnaA04g12700.1D2	RLK	variable	15	11	26
BnaA04g13170.1D2	RLK	variable	26	6	32
BnaA04g14730.1D2	RLK	variable	3	3	6
BnaA04g25370.1D2	RLP	variable	13	7	20
BnaA04g25580.1D2	RLK	variable	3	3	6
BnaA04g25730.1D2	RLK	variable	16	13	29
BnaA04g26210.1D2	RLK	variable	2	1	3
BnaA05g02280.1D2	RLK	variable	3	3	6
BnaA05g03320.1D2	RLK	variable	6	4	10
BnaA05g07850.1D2	CN	variable	1	1	2

BnaA05g07930.1D2	CN	variable	1	1	2
BnaA05g12520.1D2	TNL	variable	1	1	2
BnaA05g20760.1D2	RLK	variable	7	2	9
BnaA05g34010.1D2	RLK	variable	3	2	5
BnaA05g34470.1D2	RLK	variable	19	10	29
BnaA06g20560.1D2	RLK	variable	4	2	6
BnaA06g21980.1D2	RLK	variable	5	1	6
BnaA06g30810.1D2	CN	variable	10	2	12
BnaA06g30820.1D2	CN	variable	3	2	5
BnaA06g36910.1D2	RLK	variable	7	1	8
BnaA06g37480.1D2	RLK	variable	3	1	4
BnaA06g37520.1D2	RLK	variable	3	2	5
BnaA07g21550.1D2	NL	variable	11	4	15
BnaA07g25600.1D2	RLK	variable	2	0	2
BnaA07g30320.1D2	RLK	variable	3	2	5
BnaA07g33860.1D2	RLK	variable	4	4	8
BnaA07g34310.1D2	NL	variable	4	2	6
BnaA07g34390.1D2	RLK	variable	12	6	18
BnaA07g34700.1D2	RLK	variable	16	4	20
BnaA08g07830.1D2	RLK	variable	5	2	7
BnaA08g08200.1D2	TX	variable	2	1	3
BnaA08g14870.1D2	RLK	variable	18	6	24
BnaA08g14950.1D2	TNL	variable	1	0	1
BnaA08g19560.1D2	TNL	variable	6	4	10
BnaA08g19570.1D2	TX	variable	1	1	2
BnaA09g01450.1D2	RLK	variable	18	11	29
BnaA09g02200.1D2	RLK	variable	13	11	24
BnaA09g02790.1D2	RLK	variable	3	1	4
BnaA09g03340.1D2	RLK	variable	3	2	5
BnaA09g03980.1D2	RLK	variable	26	21	47
BnaA09g06730.1D2	RLK	variable	16	13	29
BnaA09g07840.1D2	RLP	variable	2	1	3
BnaA09g08110.1D2	NL	variable	7	3	10
BnaA09g14610.1D2	NL	variable	43	9	52
BnaA09g16950.1D2	TX	variable	4	3	7
BnaA09g16960.1D2	TX	variable	18	7	25
BnaA09g22200.1D2	RLK	variable	6	1	7
BnaA09g45960.1D2	RLK	variable	9	5	14
BnaA09g46120.1D2	RLK	variable	21	7	28
BnaA09g47750.1D2	RLK	variable	26	6	32
BnaA09g48790.1D2	RLK	variable	1	1	2
BnaA09g51310.1D2	RLK	variable	33	22	55
BnaA10g03460.1D2	RLK	variable	5	4	9
BnaA10g11120.1D2	RLK	variable	10	5	15
BnaA10g12510.1D2	RLK	variable	7	3	10
BnaA10g17850.1D2	TX	variable	2	0	2
BnaA10g17860.1D2	TX	variable	2	0	2
BnaA10g24980.1D2	RNL	variable	14	7	21
BnaC01g00150.1D2	RLK	variable	5	3	8
BnaC01g01280.1D2	RLK	variable	3	0	3
BnaC01g02060.1D2	RLK	variable	14	10	24
BnaC01g04240.1D2	RLK	variable	7	7	14
BnaC01g05390.1D2	RLK	variable	44	16	60

BnaC01g05400.1D2	RLK	variable	23	11	34
BnaC01g05460.1D2	RLK	variable	14	10	24
BnaC01g06210.1D2	RLK	variable	30	19	49
BnaC01g06760.1D2	RLK	variable	1	0	1
BnaC01g09670.1D2	RLK	variable	8	4	12
BnaC01g10040.1D2	RLK	variable	2	2	4
BnaC01g10590.1D2	NL	variable	31	14	45
BnaC01g10600.1D2	NL	variable	51	21	72
BnaC01g11510.1D2	RLK	variable	4	2	6
BnaC01g12210.1D2	RLK	variable	9	6	15
BnaC01g13830.1D2	TX	variable	1	0	1
BnaC01g13840.1D2	TX	variable	12	2	14
BnaC01g13850.1D2	TX	variable	19	8	27
BnaC01g13860.1D2	TX	variable	67	22	89
BnaC01g14230.1D2	RLK	variable	3	2	5
BnaC01g17680.1D2	CNL	variable	5	1	6
BnaC01g22970.1D2	NL	variable	14	2	16
BnaC01g24110.1D2	RLK	variable	1	0	1
BnaC01g25630.1D2	CN	variable	7	1	8
BnaC01g25650.1D2	NL	variable	10	2	12
BnaC01g26300.1D2	RLK	variable	2	1	3
BnaC01g29560.1D2	RLK	variable	10	4	14
BnaC01g30390.1D2	RLK	variable	2	0	2
BnaC01g31150.1D2	RLK	variable	3	0	3
BnaC01g33680.1D2	RLK	variable	8	2	10
BnaC01g34580.1D2	RLK	variable	25	6	31
BnaC01g34820.1D2	RLK	variable	2	1	3
BnaC01g34860.1D2	RLK	variable	9	5	14
BnaC02g05600.1D2	RLK	variable	3	3	6
BnaC02g08640.1D2	RLK	variable	1	0	1
BnaC02g08820.1D2	RLK	variable	49	27	76
BnaC02g09410.1D2	NL	variable	29	6	35
BnaC02g11050.1D2	RLK	variable	4	3	7
BnaC02g11810.1D2	RLK	variable	2	0	2
BnaC02g13840.1D2	RLK	variable	16	6	22
BnaC02g14300.1D2	RLK	variable	3	1	4
BnaC02g14380.1D2	RLK	variable	16	10	26
BnaC02g15520.1D2	RLK	variable	17	8	25
BnaC02g15560.1D2	RLK	variable	22	10	32
BnaC02g17220.1D2	OTHER	variable	22	10	32
BnaC02g17480.1D2	RLK	variable	9	2	11
BnaC02g18480.1D2	RLK	variable	1	0	1
BnaC02g19600.1D2	RLK	variable	1	1	2
BnaC02g20280.1D2	RLK	variable	29	7	36
BnaC02g20400.1D2	RLK	variable	3	3	6
BnaC02g20500.1D2	RLK	variable	1	0	1
BnaC02g21930.1D2	TNL	variable	41	12	53
BnaC02g21940.1D2	TX	variable	3	0	3
BnaC02g21970.1D2	TX	variable	23	5	28
BnaC02g21980.1D2	NBS	variable	11	4	15
BnaC02g23040.1D2	RLK	variable	5	1	6
BnaC02g24260.1D2	RLK	variable	6	4	10
BnaC02g26050.1D2	RLK	variable	5	1	6

BnaC02g26620.1D2	RLK	variable	1	1	2
BnaC02g28820.1D2	RLK	variable	31	7	38
BnaC02g29710.1D2	TX	variable	8	3	11
BnaC02g29800.1D2	RLP	variable	18	4	22
BnaC02g29850.1D2	TX	variable	10	4	14
BnaC02g30110.1D2	TX	variable	13	4	17
BnaC02g30120.1D2	TNL	variable	30	12	42
BnaC02g30660.1D2	RLK	variable	11	2	13
BnaC02g30900.1D2	RLP	variable	6	0	6
BnaC02g33190.1D2	RLK	variable	5	2	7
BnaC02g34610.1D2	RLK	variable	2	2	4
BnaC02g34790.1D2	RLK	variable	5	3	8
BnaC02g36600.1D2	RLK	variable	5	3	8
BnaC02g37300.1D2	RLK	variable	4	1	5
BnaC03g00210.1D2	RLK	variable	8	2	10
BnaC03g00890.1D2	RLK	variable	4	4	8
BnaC03g02710.1D2	RLK	variable	7	2	9
BnaC03g03570.1D2	RLK	variable	5	2	7
BnaC03g03940.1D2	RLK	variable	12	8	20
BnaC03g03960.1D2	RLK	variable	10	1	11
BnaC03g04820.1D2	RLK	variable	4	3	7
BnaC03g05760.1D2	RLP	variable	9	5	14
BnaC03g07660.1D2	TNL	variable	23	11	34
BnaC03g07670.1D2	TNL	variable	40	14	54
BnaC03g10220.1D2	RLK	variable	4	1	5
BnaC03g10290.1D2	RLK	variable	8	1	9
BnaC03g11340.1D2	RLK	variable	5	2	7
BnaC03g13010.1D2	RLK	variable	11	7	18
BnaC03g14210.1D2	RLK	variable	3	2	5
BnaC03g16790.1D2	TNL	variable	32	11	43
BnaC03g17210.1D2	RLK	variable	7	4	11
BnaC03g25970.1D2	OTHER	variable	40	13	53
BnaC03g27690.1D2	RLK	variable	3	1	4
BnaC03g47190.1D2	RLK	variable	9	3	12
BnaC04g19990.1D2	TX	variable	4	1	5
BnaC04g29920.1D2	TN	variable	9	2	11
BnaC04g33020.1D2	RLK	variable	26	6	32
BnaC05g01680.1D2	RLK	variable	2	0	2
BnaC05g34570.1D2	RLP	variable	10	3	13
BnaC05g35480.1D2	RLK	variable	13	4	17
BnaC06g17780.1D2	RLK	variable	1	0	1
BnaC06g20050.1D2	TX	variable	14	7	21
BnaC06g20380.1D2	TN	variable	10	2	12
BnaC06g20390.1D2	TX	variable	5	3	8
BnaC06g21390.1D2	RLK	variable	16	2	18
BnaC06g28360.1D2	RLK	variable	15	4	19
BnaC06g30860.1D2	RLK	variable	28	7	35
BnaC06g33940.1D2	RLK	variable	2	1	3
BnaC06g34830.1D2	RLK	variable	17	11	28
BnaC07g06940.1D2	RLK	variable	5	2	7
BnaC07g14020.1D2	RN	variable	16	3	19
BnaC07g16180.1D2	RLK	variable	29	15	44
BnaC07g20550.1D2	OTHER	variable	13	6	19

BnaC07g20570.1D2	RNL	variable	23	8	31
BnaC07g20580.1D2	OTHER	variable	44	8	52
BnaC07g25820.1D2	RLK	variable	2	0	2
BnaC07g31050.1D2	TNL	variable	25	4	29
BnaC07g31080.1D2	NL	variable	24	16	40
BnaC07g31580.1D2	RLK	variable	1	1	2
BnaC07g39900.1D2	TX	variable	3	2	5
BnaC08g05060.1D2	RLK	variable	11	1	12
BnaC08g30190.1D2	RLK	variable	4	0	4
BnaC08g32210.1D2	RLK	variable	5	1	6
BnaC08g33190.1D2	NL	variable	13	5	18
BnaC08g33210.1D2	NBS	variable	3	1	4
BnaC08g34760.1D2	RLP	variable	35	14	49
BnaC08g37150.1D2	RLK	variable	11	4	15
BnaC08g37310.1D2	RLK	variable	3	2	5
BnaC09g01240.1D2	RLK	variable	8	1	9
BnaC09g01500.1D2	RLK	variable	11	3	14
BnaC09g05400.1D2	RLK	variable	9	4	13
BnaC09g06980.1D2	RLP	variable	3	2	5
BnaC09g07280.1D2	NL	variable	8	3	11
BnaC09g07300.1D2	RLK	variable	24	9	33
BnaC09g07460.1D2	RNL	variable	18	6	24
BnaC09g08700.1D2	RLK	variable	7	2	9
BnaC09g11040.1D2	RLK	variable	6	3	9
BnaC09g14090.1D2	CNL	variable	23	6	29
BnaC09g17130.1D2	NBS	variable	5	2	7
BnaC09g17190.1D2	TNL	variable	34	16	50
BnaC09g17200.1D2	TNL	variable	25	7	32
BnaC09g17220.1D2	NL	variable	2	1	3
BnaC09g17230.1D2	TX	variable	1	0	1
BnaC09g17240.1D2	NL	variable	1	1	2
BnaPanG000207.1D2	RLK	variable	14	10	24
BnaPanG000231.1D2	RLK	variable	4	1	5
BnaPanG000354.1D2	NBS	variable	4	2	6
BnaPanG000488.1D2	RLK	variable	16	3	19
BnaPanG000544.1D2	RLP	variable	2	0	2
BnaPanG000555.1D2	NL	variable	22	10	32
BnaPanG000556.1D2	TNL	variable	29	9	38
BnaPanG000645.1D2	RLP	variable	12	9	21
BnaPanG000701.1D2	RLK	variable	23	8	31
BnaPanG000750.1D2	RLP	variable	11	1	12
BnaPanG000830.1D2	NL	variable	1	0	1
BnaPanG000848.1D2	RLP	variable	2	0	2
BnaPanG000919.1D2	RLK	variable	9	4	13
BnaPanG001091.1D2	RLK	variable	8	3	11
BnaPanG001152.1D2	RLP	variable	20	6	26
BnaPanG001156.1D2	NL	variable	2	0	2
BnaPanG001158.1D2	RLK	variable	3	2	5
BnaPanG001326.1D2	NL	variable	6	4	10
BnaPanG001380.1D2	NL	variable	2	0	2
BnaPanG001423.1D2	RLK	variable	14	7	21
BnaPanG001558.1D2	NL	variable	5	2	7
BnaPanG001582.1D2	RLK	variable	1	0	1

BnaPanG001674.1D2	RLP	variable	5	2	7
BnaPanG001865.1D2	RLP	variable	19	5	24
BnaPanG002126.1D2	RLP	variable	14	5	19
BnaPanG002127.1D2	NL	variable	18	9	27
BnaPanG002466.1D2	RLK	variable	4	2	6
BnaPanG002561.1D2	NBS	variable	5	2	7
BnaPanG002676.1D2	NL	variable	17	8	25
BnaPanG002706.1D2	RLP	variable	11	5	16
BnaPanG003414.1D2	TNL	variable	31	11	42
BnaPanG004341.1D2	RLP	variable	47	19	66
BnaPanG004913.1D2	RLP	variable	7	1	8
BnaPanG005274.1D2	RLP	variable	5	2	7
BnaPanG005637.1D2	NBS	variable	12	1	13
BnaPanG008701.1D2	RLK	variable	13	5	18
BnaPanG011391.1D2	NL	variable	14	4	18
BnaU01g03560.1D2	RLK	variable	2	0	2
BnaU01g04360.1D2	TX	variable	6	3	9
BnaU01g04860.1D2	RLK	variable	1	1	2
BnaU01g09120.1D2	RLK	variable	1	0	1
BnaU01g09230.1D2	TX	variable	3	0	3
BnaU01g13700.1D2	RLK	variable	13	7	20
BnaU01g16220.1D2	RLK	variable	3	0	3
BnaU01g20130.1D2	RLK	variable	2	0	2
BnaU01g22020.1D2	RLK	variable	6	4	10
BnaU01g23500.1D2	RLK	variable	2	1	3
BnaU01g23510.1D2	RLK	variable	10	4	14
		**Total**	**3,348**	**1,386**	**4,734**
		**Grand total**	**10,375**	**4,943**	**15,318**

Supplementary Table 6: The numbers of non-synonymous and synonymous SNPs, missense and nonsense variants and other effects in different RGAs

RGAs	Number of RGAs with SNP	Non-synonymous SNPs	Synonymous SNPs	Total SNP	Missense	Nonsense	Other effects
CN	13	71	25	96	45	0	1
CNL	12	146	44	190	93	1	12
NBS	16	142	50	192	86	1	7
NL	59	969	383	1352	569	5	24
OTHER	14	286	103	389	174	3	13
RN	3	46	20	66	23	1	3
RNL	6	123	46	169	69	1	9
TN	12	105	39	144	62	3	3
TNL	26	682	237	919	416	9	29
TX	46	383	135	518	234	4	11
**Total**	**207**	**2,953**	**1,082**	**4,035**	**1,771**	**28**	**112**
RLP	65	639	259	898	332	9	52
RLK	758	6,783	3,602	10,385	2,810	49	426
**Total**	**823**	**7,422**	**3,861**	**11,283**	**3,142**	**58**	**478**
**Grand total**	**1030**	**10,375**	**4,943**	**15,318**	**4,913**	**86**	**590**

Supplementary Table 7: RGA candidates underlying reported QTL for blackleg in the Darmor v 8.1 assembly

Locus	Marker	QTL reference	Start (Mbp)	End (Mbp)	Length (Mbp)	RGA candidates	Core genes percentage
Rlm1	Na12A02<br>Ol12-E03A<br>CB10544A<br>Ra2-A05b<br>BSR+KASP	-<br>Delourme et al., 2004<br>Raman et al., 2012b<br>-	22.35	27.29	4.94	RLK 17, TX 1	16 core, 2 variable (88.88% core)
Rlm3	BnGMS147b<br>IGF0504f_F	Delourme et al., 2004<br>Leflon et al., 2007	8.97	25.75	16.79	RLK 38, RNL 2, RLP 3, TX 2, NL 1	43 core 3 variable (93.47% core)
Rlm4	BRMS040<br>BRAS023<br>BRMS005b<br>Na12-E11b<br>Ol09-A06<br>BRMS075<br>CB10278a<br>KBRH143H15<br>BRMS036<br>Bn204<br>Na12-A02<br>Ra2-A05	Raman et al., 2012a	1.63	28.32	26.69	RLK 51, RNL 2, RLP 3, TX 2, NL 2	53 core, 7 variable (88.33% core)
	DBSNP10503<br>DBSNP10504<br>DBSNP31606<br>DBSNP30220<br>DBSNP01654<br>DBSNP01910<br>DBSNP05704<br>DBSNP05705	Tang and Zhao, 2015					
Rlm7	sR7018	Larkan et al., 2016	12.16	28.19	16.02	RLK 38, RNL 2, RLP 3, TX 2, NL 1	42 core, 4 variable (91.30% core)
Rlm9	BnGMS665<br>BnGMS147b	Delourme et al., 2004	13.76	19.11	5.35	RLK 13, RNL 2, RLP 2	17 core (100% core)
LepR1	Flanking Markers	Larkan et al., 2016	10.02	20.43	10.41	RLK 13, NL 1	12 core, 2 variable (85.71% core)
LepR2	Flanking Markers	Larkan et al., 2016	0.20	14.15	13.95	RLK 29, TN 2, OTHER 1	28 core, 4 variable (87.50% core)
